# A receptor-mediated landscape of druggable and targeted nanomaterials for gliomas

**DOI:** 10.1016/j.mtbio.2023.100671

**Published:** 2023-05-19

**Authors:** Leonardo Delello Di Filippo, Suzana Gonçalves de Carvalho, Jonatas Lobato Duarte, Marcela Tavares Luiz, Jessyca Aparecida Paes Dutra, Geanne Aparecida de Paula, Marlus Chorilli, João Conde

**Affiliations:** a School of Pharmaceutical Sciences, São Paulo State University (UNESP), Araraquara, São Paulo, Brazil; b ToxOmics, NOVA Medical School, Faculdade de Ciências Médicas, NMS|FCM, Universidade NOVA de Lisboa, Lisboa, Portugal

**Keywords:** Molecular machinery, Glioma, Brain cancer, Target delivery, Drug delivery, Nanomedicine

## Abstract

Gliomas are the most common type of brain cancer, and among them, glioblastoma multiforme (GBM) is the most prevalent (about 60% of cases) and the most aggressive type of primary brain tumor. The treatment of GBM is a major challenge due to the pathophysiological characteristics of the disease, such as the presence of the blood-brain barrier (BBB), which prevents and regulates the passage of substances from the bloodstream to the brain parenchyma, making many of the chemotherapeutics currently available not able to reach the brain in therapeutic concentrations, accumulating in non-target organs, and causing considerable adverse effects for the patient. In this scenario, nanocarriers emerge as tools capable of improving the brain bioavailability of chemotherapeutics, in addition to improving their biodistribution and enhancing their uptake in GBM cells. This is possible due to its nanometric size and surface modification strategies, which can actively target nanocarriers to elements overexpressed by GBM cells (such as transmembrane receptors) related to aggressive development, drug resistance, and poor prognosis. In this review, an overview of the most frequently overexpressed receptors in GBM cells and possible approaches to chemotherapeutic delivery and active targeting using nanocarriers will be presented.

## Graphical abstract

**Figure:**
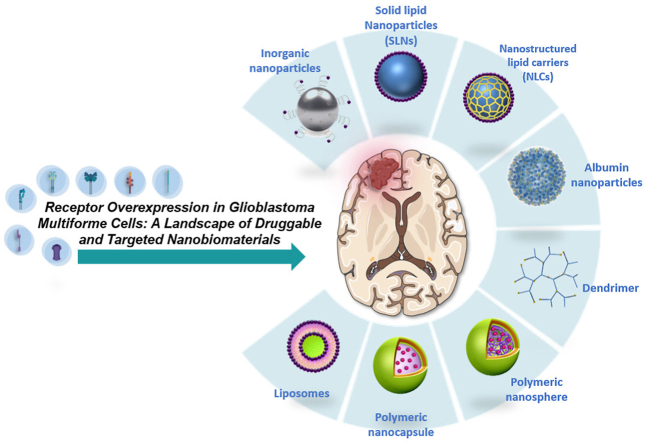

## 1 Introduction

### 1.1 Glioblastoma multiforme

Gliomas are brain tumors with a frequency of 80% of all malignant brain cancers [1]. Among them, glioblastoma multiforme (GBM), a grade IV intracranial astrocytoma, is the most common primary brain tumor in adults, responsible for around 60% of all gliomas [2].

One of the key features of GBM is the presence of genetic mutations that promote the growth and survival of cancer cells. Mutations in genes and upregulation of the p53 tumor suppressor pathway, the RB (retinoblastoma protein) pathway, and the PI3K/AKT/mTOR pathway are commonly found in GBM. These mutations affect several important cellular pathways, including cell cycle regulation, DNA repair, and signal transduction. As a result, GBM cells can divide rapidly and evade apoptosis (cell death), leading to uncontrolled growth and tumor formation. Epigenetic changes, which alter gene expression without changing the underlying DNA sequence, are also important in GBM. For example, DNA methylation and histone modification can silence tumor suppressor genes and promote the expression of oncogenes, leading to the development and progression of GBM. Additionally, miRNAs (micro intefering RNA molecules that regulate gene expression) are dysregulated in GBM and can affect key signaling pathways, also leading to tumor progression. The tumor microenvironment plays a critical role in GBM biology as well. GBM tumors are characterized by abnormal blood vessels and hypoxic (low oxygen) conditions, which contribute to tumor growth and resistance to drugs. Immune cells in the tumor microenvironment, such as T-lymphocytes and macrophages, can also promote tumor growth and immunosuppression by modulating the cytokines secretion into a pro-inflammatory profile [3].

Finally, GBM tumors are highly heterogeneous, meaning that different regions of the tumor can have distinct genetic and molecular profiles. This heterogeneity contributes to therapy resistance and tumor recurrence, as some regions of the tumor may be more resistant to treatment than others, besides the presence of GBM stem cells (GBMSCs). GBMSCs have several characteristics that distinguish them from other cancer cells in the tumor. They can self-renew and differentiate into multiple cell types within the tumor, contributing to tumor heterogeneity. They also have increased resistance to chemotherapy and radiation therapy, which can allow them to survive and contribute to tumor recurrence. These stem cells can initiate and maintain the tumor, and are suggested to be responsible for the rapid growth and infiltrative nature of GBM tumors [4].

**Fig. 1:**
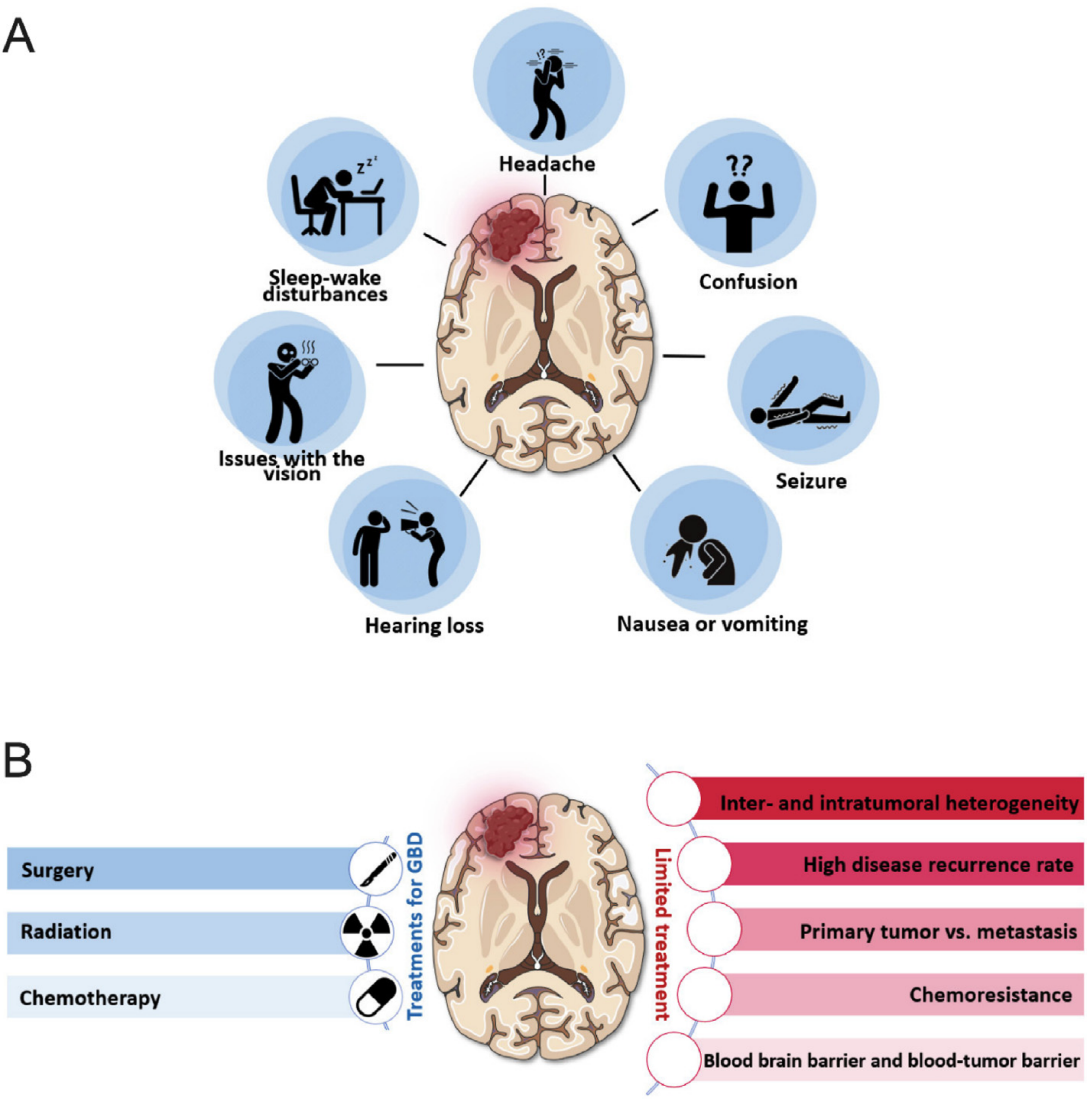
**A** - Clinical symptoms often associated with GBM. Patients may experience headaches, confusion, seizures, nausea or vomiting, hearing loss, vision impairment, and sleep disturbances. 1-**B -** Clinical management of GBM currently requires a multidisciplinary approach involving surgical resection of the tumor, in addition to adjuvant radiotherapy and chemotherapy. Treatment of GBM is still a challenge due to the biological characteristics of this tumor, which include tumor heterogeneity, high recurrence rates, the development of secondary gliomas, chemoresistance, and the presence of the BBB, which regulates and prevents the penetration of many chemotherapeutic drugs into the brain.

About 90% of GBM cases are a primary tumor, most often affecting the age group of 60–64 years or older, while the remaining cases develop from secondary gliomas (resulting from a low-grade astrocytoma), with a higher incidence in individuals aged 40–50 years [1,5,6]. GBM affects more men than women (1.6:1), as well as Caucasian populations [7]. Clinical manifestations range from headaches and neurological deficits to seizures, depending on the location and size of the tumor in the brain (Fig. 1-A). The diagnosis is made through tests to confirm the condition, which are diagnosed through laboratory tests associated with imaging techniques, such as magnetic resonance and/or computed tomography [[8], [9], [10]].

Once the diagnosis is defined, multimodal therapy is initiated, recommending surgical removal of the tumor's greatest possible extension, in addition to adjuvant radiotherapy and chemotherapy (Fig. 1-B). However, even when combining three different therapeutic modalities, the median survival does not exceed 15 months from diagnosis [11]. Thus, the search for new therapies against GBM is greatly encouraged. The pharmacotherapy of GBM is a particular challenge because of its pathophysiological characteristics, its intracranial location, and the presence of the blood-brain barrier (BBB), which prevents and regulates the passage of substances from the bloodstream to the brain parenchyma [12].

Additionally, evidence shows that GBM has become resistant to the drugs currently used, with emphasis on temozolomide (TMZ), the first-line drug, leading to the need to increase the dose, which consequently generates toxicity and adverse events, compromising the patient's life quality and chemotherapy treatment in general [13]. In recent decades, the need for new technologies with biopharmaceutical properties suitable for the treatment of GBM has become evident, with formulations capable of crossing the BBB, selectively targeting the tumor tissue, and efficiently eliminating tumor cells only [[14], [15], [16], [17]].

In this context, the present article aims to provide an overview of the main receptors overexpressed in GBM cells, highlight their role in the development of this neoplasm, and discuss their use as possible therapeutic targets. Studies from the last 10 years that use molecules to block these receptors or that are actively targeted through surface modification will be considered. This work aims to promote and advance the understanding of new therapeutic approaches with superior efficacy, better brain bioavailability, and lower systemic biodistribution (to reduce adverse effects and toxicity) toward a more efficient and safe treatment for GBM.

### 1.2 GBM standard-of-care therapy

Temozolomide (TMZ) is a prodrug (Fig. 3), which is stable in acidic pH but hydrolyzes in its active form at pH greater than 7.0, making it the chemotherapy of choice for GBM treatment [18]. It is a low molecular weight imidazole derivative (194.15 ​Da), available orally, capable of crossing the blood-brain barrier (BBB) and reaching the Central Nervous System (CNS) due to its lipophilicity [[18], [19], [20]]. Its maximum plasma concentration is around 1 ​h after oral administration, with a short half-life of only 1.8 ​h and almost complete elimination of plasma within 8 ​h [21].

**Fig. 2:**
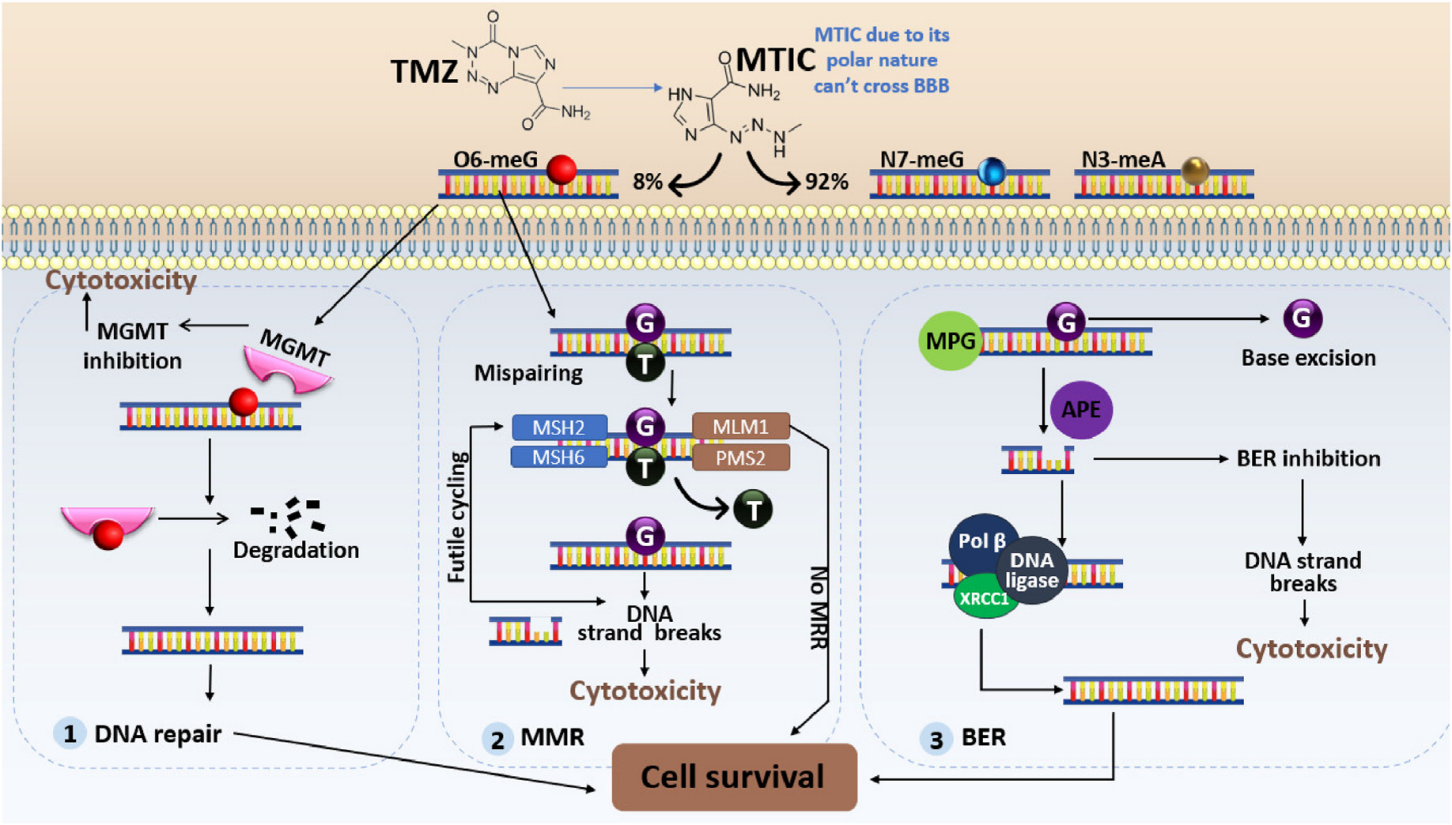
Biological metabolism of TMZ into its active derivative, MTIC, and known cellular resistance mechanisms involved in the resistance of GBM cells to TMZ. One of the primary mechanisms involves the expression of the DNA repair protein O6-methylguanine-DNA methyltransferase (MGMT). MGMT repairs the DNA damage caused by TMZ by directly removing the alkyl group from the O6 position of guanine residues, thereby preventing the formation of lethal DNA adducts. The Mismatch Repair (MMR) system is a tumor suppressor mechanism that corrects base substitution mutations and small insertions/deletions. Loss of MMR capacity can mediate resistance to TMZ because O6-methylguanine (O6-MeG) is recognized by MMR and the thymine residue is removed. However, in the absence of MGMT, O6-MeG remains, and the thymine is reinserted in the opposite way to O6-MeG, resulting in gaps in the synthesized DNA that lead to cell death. The Base excision repair (BER) system repairs modifications of DNA bases caused by chemical agents, including TMZ-induced N7MG adducts. AAG recognizes and removes the modified base, creating an apurinic site. APE1 cleaves the damaged end, and DNA polymerase β (Pol β) fills the gap with a single nucleotide. DNA ligase I or a complex of XRCC1 and DNA ligase III seals the nick. The up-regulation of AAG and inhibition of DNA Pol β can increase the sensitivity of GBM cells to TMZ.

TMZ works by alkylating purine DNA bases and adding a methyl group to the sites O6 and N7 of guanine and N3 of adenine (Fig. 2) [20,22]. N-methylation is typically reversed by basic excision repair enzymes, while methylation of guanine site O6 is responsible for the therapeutic effect of TMZ [23]. O6-methylguanine promotes nucleotide incompatibility in the next DNA replication cycles, leading to the replacement of cytosine by thymine [20]. To remove this methylated adduct, the cell repair mechanism (MRR) causes DNA damage, leading to cell cycle arrest and consequent apoptosis [20,21].

**Fig. 3:**
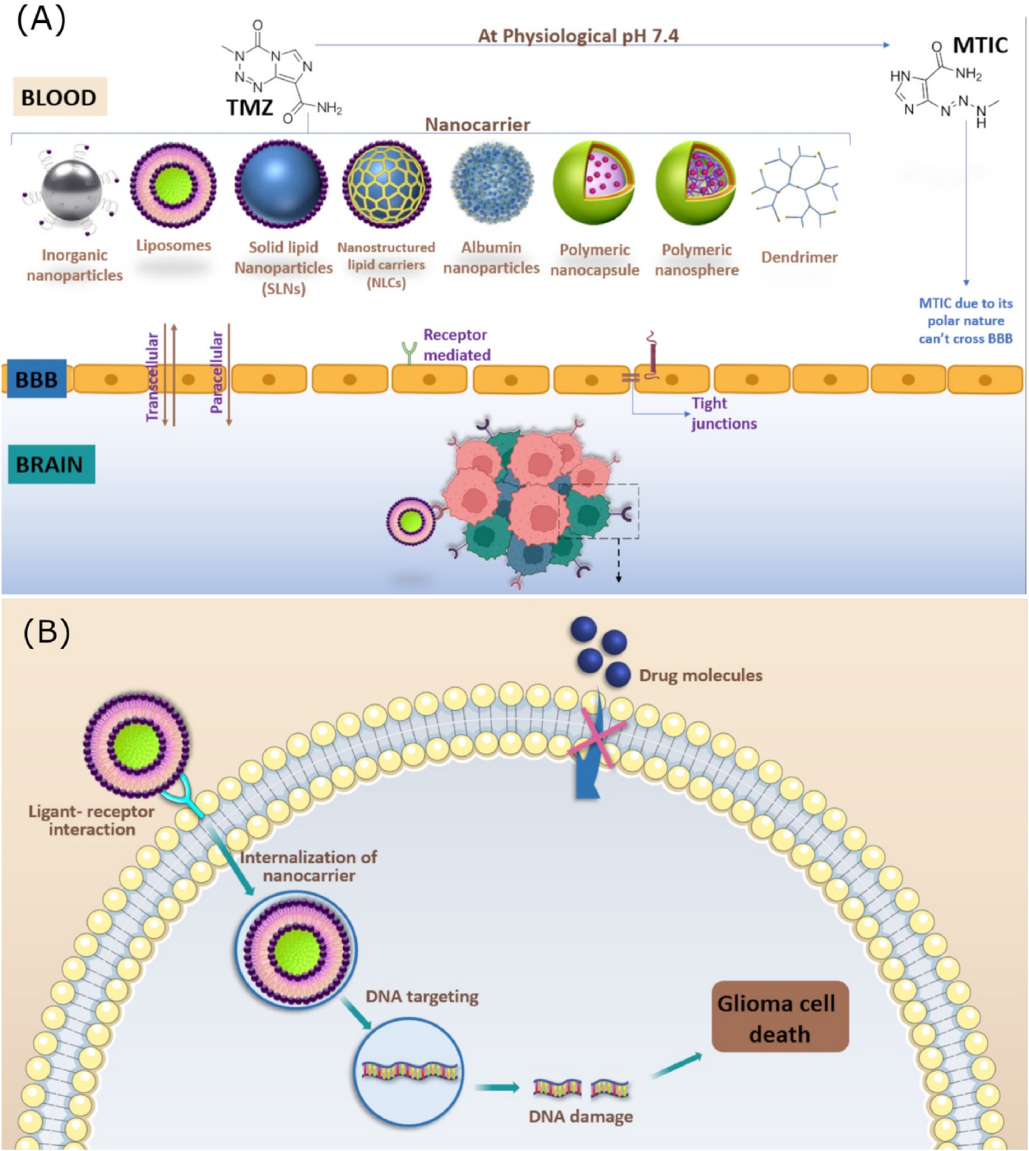
Many drugs such as temozolomide, bevacizumab, lomustine and carmustine have promising antitumor activity, but their brain bioavailability is limited by their physicochemical characteristics and/or by the presence of the BBB, as in the case of TMZ. A variety of nanocarriers of inorganic, polymeric, or lipid nature can be used to improve the transposition of the BBB, either by their passive accumulation due to their small size or through their active uptake, made possible by the surface modification of the nanocarriers (A). These strategies are also capable of improving interaction with neoplastic cells, facilitating their internalization, and enhancing their anti-tumor effect (B).

The patient treated with TMZ normally receives high doses of the drug in order to achieve the therapeutic effect, and this extensive exposure to the drug, combined with the heterogeneity of the tumor, favor the development of resistance to TMZ [24]. The understanding of resistance mechanisms, however, may be somewhat limited, as these can be acquired during treatment or inherent to the tumor [25].

One of the advantages of TMZ in the treatment of GBM is its greater ability to cross the BBB compared to other alkylating agents. Thus, only a small fraction of the drug can cross this barrier and reach its site of action, making it necessary to administer high doses of TMZ, which accumulates in non-targeted tissues and causes systemic side effects [18,26,27]. In addition to functioning as a physical barrier for the passage of molecules, the BBB has an active efflux system for drugs, such as Glycoprotein-P, a membrane protein capable of expelling the drug from the tissue [27,28].

Another obstacle is the DNA repair mechanism, through which the Protein O6-methylguanine methyltransferase (MGMT) removes the alkyl groups, inserted by TMZ, from the O6 position of the guanine of DNA (Fig. 3), promoting resistance to chemotherapy [23,28,29]. MGMT is present in both the nucleus and cytoplasm of the cell and is consumed in a stochiometric reaction during the repair process, not being regenerated at the end of the reaction [21,23,29].

The therapeutic effect of TMZ depends on the action of DNA mismatch repair proteins (MMR), which identify the incorrect pairing of bases and perform cycles of excision of the poorly paired bases, leading to the breakdown of DNA tapes and cell death (Fig. 2) [27]. Thus, MMR deficiency or the presence of mutations in this protein, especially in the MSH6 subunit, which recognizes changes in O6-methylguanine, causes the cell to become resistant to the action of TMZ [20,23,27]. Still, although less significant, baseline excision (BER) repair may also be related to resistance to TMZ. BER rapidly corrects DNA damage in N7-methylguanine and N3-methylladenine adducts [20]. Therefore, mutations in BER components, such as the enzyme poly (ADP-ribose) polymerase-1 (PARP-1), responsible for the accumulation of DNA fragments in cells that leads to their death, can reduce resistance to TMZ by compromising the functioning of BER [20,29,30].

### 1.3 Materials science and brain cancer

The design of nanomaterials for drug delivery or active targeting is a complex process that requires careful consideration of the target location, desired function, and other factors such as biocompatibility and stability. If the goal is to achieve active targeting, the nanoparticles must be designed to interact specifically with the target cells or tissues. This can be achieved by incorporating ligands (such as peptides, polysaccharides, antibodies, etc) on the surface of the nanoparticles which can bind to specific receptors on the target cells [31]. On the other hand, if the goal is to deliver inhibitors, the nanoparticles must need to be designed to release their cargo in a specific manner. For example, if the target is located extracellularly (as in the case of many receptors overexpressed in the cell membrane of glioma cells), the nanoparticles may need to be designed to release their cargo once in the tumor microenvironment. This can be achieved by incorporating stimuli-responsive materials that can respond to changes in pH, temperature, or other environmental factors [32]. The same logic is valid for drugs that must be delivered in the cell cytoplasm. In addition, the size and shape of the nanoparticles can also play a role in their biopharmaceutical properties. For example, smaller nanoparticles may be able to penetrate deeper into tissues, while larger nanoparticles may be better suited for targeting specific cells or organs [33].

**Fig. 4:**
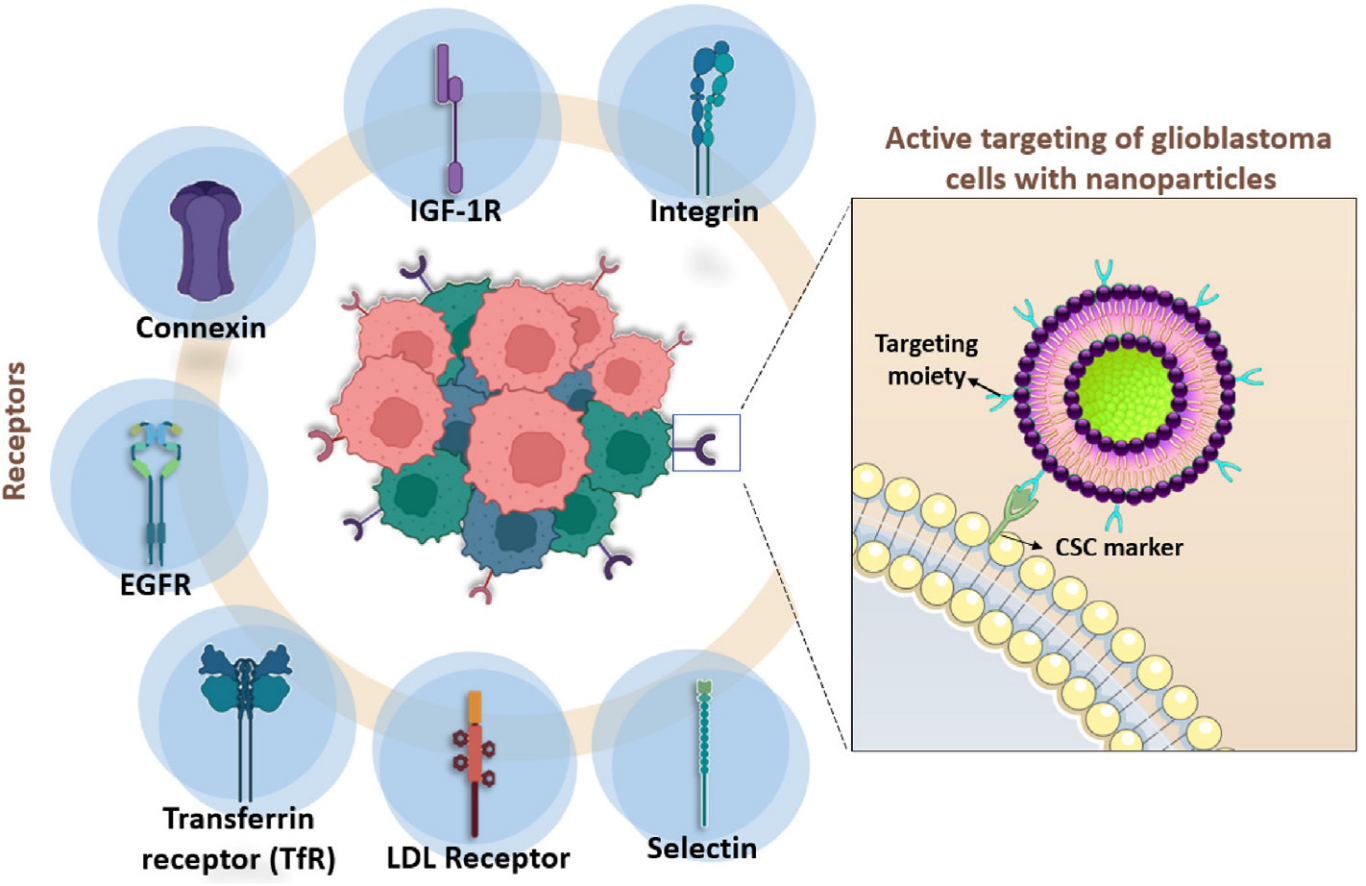
Gliomas overexpress various receptors, such as integrin, IGF-1R, connexin, EGFR, transferrin, LDL receptors, and selectin. These receptors are involved in critical biological functions, including cell adhesion, growth, and proliferation, and have been identified as promising targets for the delivery of nanoparticles to gliomas. By conjugating nanoparticles with ligands that can bind to these receptors, active targeting can increase the accumulation of nanoparticles in gliomas, resulting in enhanced therapeutic efficacy and reduced systemic toxicity.

In this context, pharmaceutical nanotechnology emerged as a promising approach to improve GBM treatment. The use of nanostructured systems (Fig. 4) for drug delivery improves the bioavailability of this drug in the brain, by facilitating its permeation through the BBB due to its nanometric size and appropriate surface charge, as well as to surface modifications that facilitate the active transport across the BBB (Fig. 1) [34].

The blood-brain barrier (BBB) is a highly selective semipermeable structure composed of blood vessels and glial cells that separates the circulating blood from the brain's extracellular fluid. The BBB is critical for maintaining a stable environment in the brain and protecting it from harmful substances. However, the BBB also represents a significant challenge for delivering drugs to the CNS, as many drugs – such as chemotherapeutics, cannot cross the BBB in desirable concentrations for therapeutic activity. In this context, nanoparticles can be particularly useful to deliver drugs to the CNS due to their distinct properties that make them efficient vehicles with an increased ability to cross the BBB [35].

The size of the nanoparticles plays a crucial role in their ability to penetrate the BBB. Smaller nanoparticles are preferable for BBB permeation through opened gaps in the BBB, but nanoparticles in the single-nm range are quickly removed from the bloodstream via the kidneys [36]. The shape, and flexibility can significantly impact their ability to interact with the BBB. For example, experiments have demonstrated that endothelial association and basolateral transport are two coupled yet distinct processes, which can be influenced by nanoparticle properties. While smaller polystyrene (PS) spheres are better at endothelial association with decreasing size, transport is optimal for 200 ​nm spheres, suggesting that 100 ​nm spheres accumulate on or within the endothelium. PS rods associate with the endothelium significantly less than spheres but have similar transport rates. Stiff spheres both associate with endothelial cells and are transported through them much more than their soft counterparts [37].

In addition to their size, these nanocarriers can be designed with specific properties that enable them to interact with the BBB and facilitate drug delivery. For example, some nanoparticles can be coated with molecules that interact with natural receptors on the BBB, which allows them to bind to and cross the barrier more easily. Additionally, nanocarriers can be engineered to release their cargo in response to specific triggers, such as changes in pH or temperature, which can improve drug delivery to the brain. Another important property that facilitates the permeation through BBB is the nanoparticles’ surface charge; current literature reports that positively charged nanocarriers can cross the BBB more easily due to the adsorptive-mediated transcytosis pathway, resulting from the interaction between the positive charge from nanoparticles and the negatively charged luminal membrane of the brain endothelial cells [38].

Furthermore, nanomaterials can also protect drugs from degradation and clearance by the body, allowing them to reach the brain in higher concentrations. They can also target specific cells or tissues in the brain, through surface modification, further enhancing drug delivery. This technique can be useful to target elements present in the glioma cells or the tumor microenvironment [39].

GBM is one of the most vascularized human cancers [40], which favors passive accumulation by the enhanced permeability and retention (EPR) effect (nanoparticles can passively modulate the biodistribution of drugs and increase its accumulation in cancer tissues with increased vasculature permeability), which is dependent on the highly angiogenic nature of GBM where leaky vasculature is commonly present [41].

Another important mechanism is the active targeting of nanocarriers to GBM cells through surface modifications, whose targets are cellular elements overexpressed in cancer cells (i.e.: transmembrane receptors, growth factors secreted by the cell, or even elements of the extracellular matrix of these tissues) (Fig. 4). Active targeting can improve drug biodistribution by increasing the accumulation in cancer cells through receptor-mediated endocytosis; such alterations in the cell machinery occur due to molecular alterations in GBM and are related to aggressive characteristics of development and resistance to chemotherapeutics [17,42,43].

The molecular classification of gliomas is a robust tool to determine genomic alterations linked to mechanisms of tumorigenesis, growth, and drug resistance. Currently, several molecular alterations characteristic of GBM are known, related to dysregulation in the receptor tyrosine kinases (RTKs)/Ras/phosphatidylinositol 3-kinase (PI3K) pathway, which is altered in 88% of patients, with overexpression of EGFR, PDGFR, and VEGFR. Other important receptors are also overexpressed in GBM cells and related to poor prognosis, such as folate receptors, transferrin receptors and integrins. Understanding how these mechanisms are affected and how they relate to the aggressive development and drug resistance of GBM allows a more assertive treatment choice according to the individual response of each tumor to treatments and the development of more specific and safe therapies for GBM [17,44,45].

## 2 Receptor-mediator targeting

### 2.1 Receptor tyrosine kinase (RTK)

**Fig. 5:**
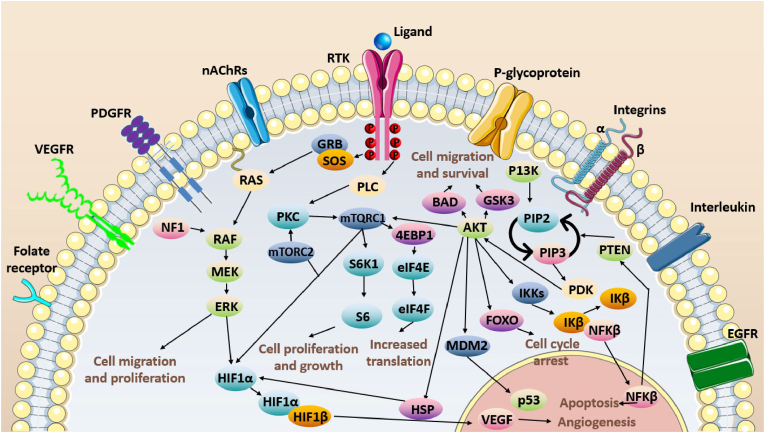
The extracellular ligand-binding domain of RTKs, which binds to a variety of ligands, leading to receptor dimerization and autophosphorylation of the tyrosine kinase domain. This autophosphorylation leads to the activation of two main downstream signaling pathways: the Ras/MAPK pathway and the PI3K/Akt/mTOR pathway. The Ras/MAPK pathway is activated by the recruitment of Grb2/SOS to the phosphorylated tyrosine residues of RTKs, which activates Ras and subsequently leads to the activation of MAPKs. This pathway is involved in cell proliferation, differentiation, and survival. The PI3K/Akt/mTOR pathway is activated by the phosphorylation of the tyrosine residues of RTKs, leading to the activation of PI3K and subsequent activation of Akt and mTOR. This pathway is involved in cell growth, survival, and metabolism.

Receptor tyrosine kinases (RTKs) are a family of membrane-spanning proteins comprising a unique ligand-binding region in the extracellular domain, a single hydrophobic transmembrane domain, and a cytoplasmatic tyrosine kinase domain [45,46]. The extracellular ligand-binding domain can be activated by many ligands, including hormones, cytokines, growth factors, neurotrophic factors, and other signaling molecules. This binding of extracellular ligands to the RTKs induces receptor dimerization and autophosphorylation of the tyrosine kinase domain, with subsequent activation of two main downstream signaling pathways: mitogen-activated protein kinase (Ras/MAPK) and phosphatidylinositol-3-kinase (PI3K/Akt/mTOR) pathways (Fig. 5) [47,48].

In the Ras/MAPK pathway, a GDP/GTP exchange regulator and a GTPase-stimulating protein mediate the signals from the kinases since the Ras protein is a GTP-binding protein. The adaptor molecules growth factor receptor-bound protein 2 (GRB2) and the guanine nucleotide exchange factor son of sevenless (SOS) recruit to the membrane and activate the RAS protein via autophosphorylation of the tyrosine domain in RTKs. The exchange of GDP for GTP activates the serine/threonine kinase Raf. Raf phosphorylates and activates MEK1/2 (also known as MAPKK1/2), which are ERK1 and ERK2 specificity kinases. ERK1/2 act as effector substrates for a variety of proteins and factors that control cell cycle progression. Raf also activates mitogen-activated protein kinase (MAPK3 or MAP3) that activates the kinases MKK4/7, MKKK3/6, and MEK5, which activate the effector pathways JNK1/2, p38, and ERK5, consecutively [[49], [50], [51]].

The PI3K/AKT/mTOR pathway is activated by the growth factor receptor tyrosine kinase that phosphorylates the phosphatidylinositol 3-kinase (PI3K). Activated PI3K is translocated to the cell membrane, where it forms phosphatidylinositol 3,4,5-triphosphate (PIP3). The serine/threonine kinase Akt/PKB is translocated to the membrane to bind to PIP3 via its pleckstrin homology (PH) domain. Akt is phosphorylated by phosphoinositide-dependent protein kinase 1 (PDK1) and the rapamycin-insensitive complex (mTORC2) or PDK2. Phosphorylated Akt is a target for several proteins involved in cell cycle regulation, such as mTORC2, forkhead-box O (FoxO) transcription factors, BCL2-associated agonist of cell death (BAD), and others. Active Akt regulates the activation of the tuberous sclerosis complex (TSC1/TSC2). TSC2 negatively regulates the Ras homolog enriched in the brain (Rheb) which is positively regulated by the rapamycin-sensitive mTOR-complex (mTORC1). The activation of mTORC1 regulates various downstream targets involved with protein synthesis, lipid synthesis, lysosome biogenesis, and autophagy. Phosphatase and Tensin Homolog (PTEN) prevent Akt translocation to the plasma membrane by dephosphorylating PIP3 [52,53].

In healthy cells, the activation of these two downstream pathways by extracellular ligands is involved in the regulation of cellular growth, differentiation, survival, and migration. However, RTKs are commonly deregulated in cancer cells and cause the aberrant activation of both downstream pathways, which have been associated with the malignancy of several solid tumors, such as breast cancer, hepatocellular carcinoma, non-small cell lung cancer, and glioblastoma multiforme [54,55].

It is estimated that RTK signaling pathways are deregulated in about 88% of patients with glioblastoma multiforme. In the human genome, 58 RTKs were identified, which are categorized into 20 classes according to the similarities in their extracellular region. The most frequent dysregulations of RTK are the amplification or mutation of epidermal growth factor receptor (EGFR), platelet-derived growth factor receptor (PDGFR), vascular endothelial growth factor receptor (VEGFR), fibroblast growth factor receptor (FGFR), and mesenchymal-epithelial transition (MET) [[55], [56], [57]]. As these RTKs and their downstream pathways play an important role in glioma malignancy and angiogenesis, they have been investigated as a potential target for glioma chemotherapy [45].

EGFR, also known as HER1 or ErBB1, belongs to a family of four RTKs: EGFR/HER1, ErbB2/HER2, ErbB3/HER3, and ErbB4/HER4 that control cell proliferation, migration differentiation, and homeostasis. The dysregulation of EGFR is frequently found in high-grade gliomas, where in 57% of cases mutations, readjustments, selective linking, and amplification can occur. The most frequent genetic alteration found in GBM (20–50%) is the EGFR variant III (EGFRvIII), which has constitutively active kinase activity in a ligand-independent manner. The overexpression of this variant has been associated with malignant progression [[58], [59], [60]]. EGFR has been the most investigated RTK due to its high frequency of dysregulation in tumors. Thus, several small-molecule inhibitors and monoclonal antibodies have been investigated for glioma treatment, such as gefitinib, erlotinib, icotinib, lapatinib, neratinib, afatinib, cetuximab, nimotuzumab, and panitumumab [31,61].

PDGFR belongs to a family of growth factor receptors consisting of PDGFRα and PDGFRβ. Platelet-derived growth factor (PDGF) ligand and its receptors are frequently overexpressed in high-grade gliomas, representing the second most frequent RTK dysregulation in GBM (about 10–13% of cases) [45,57]. It has been an attractive target in cancer therapy, given the importance of this RTK in supporting glioma genesis. Thus, several PDGRF inhibitors have been studied, including imatinib, sunitinib, sorafenib, vandetanib, and tandutinib [62].

VEGFRs are RTKs that have been identified as VEGFR-1, VEGFR-2, and VEGR-3, in which VEGFR-1 and VEGFR-2 regulate angiogenesis and VEGFR-3 regulates lymphangiogenesis [54]. Vascular endothelial growth factors (VEGF), which include VEGF-A, VEGF-B, VEGF-C, VEGF-D, VEGF-E, and placenta-like growth factor (PIGF), are responsible for activating these receptors [63,64]. In response to hypoxic conditions in the tumor microenvironment, the upregulated hypoxia-inducible transcription factors (HIF1α and HIF1β) induce the transcription of VEGF [65]. Thus, the increased activation of VEGFR by high levels of VEGF contributes to the irregular vasculature associated with gliomas. In this way, several agents with the ability to inhibit VEGF (e.g., bevacizumab and aflibercept) or its receptors (e.g., cediranib, sunitinib, sorafenib, vatalanib, vandetanib, cabozantinib, and ramucirumab) have been evaluated for glioma therapy [57,66].

FGFR is an RTK that consists of four receptors (FGFR1, FGFR2, FGFR3, and FGFR4), these receptors can be activated by 22 fibroblast growth factors (FGF) [67]. Among these receptors, FDFR1 has been associated with glioma stemness, invasion, and radioresistance, which contributes to the poor prognosis of this cancer. Furthermore, changes in the expression of FGFR in normal cells can cause malignant transformation and glioma progression owing to the activation of antiapoptotic, migratory, and mitogenic responses [45,68]. Thus, blocking FGFR signaling can be an interesting strategy for glioma treatment.

MET is an RTK well-characterized proto-oncogene that is activated by hepatocyte growth factor (HGF). In gliomas, MET plays an important role in tumor progression, drug resistance, and tumor recurrence. The expression levels of HGF and MET are frequently correlated with glioma grade [55,69]. The oncogenic activation of this receptor can result from amplification of MET, upregulation of HGF, mutations of HGF, loss of regulatory mechanisms, and constitutive kinase activity [70]. Antibodies and small molecules able to bind to MET and inhibit HGF binding have been evaluated to avoid the activation of downstream pathways responsible for tumor malignancy, including monoclonal antibodies (e.g., rilotumumab and onartuzumab), carbozantinib, foretinib, capmatinib, and volitiniv [45,57,70].

#### 2.1.1 Nanomaterials for delivering RTK inhibitors

The RTK signaling pathways are deregulated in about 88% of gliomas, which makes these receptors and their ligands potential therapeutic targets for glioma therapy. Nowadays, research has investigated the therapeutic effect of several tyrosine kinase inhibitors and molecules that block ligand binding to the receptors. The tyrosine kinase inhibitors act to avoid the autophosphorylation of RTKs, whereas other molecules can bind to the RTK extracellular domain or the receptor's ligand and prevent ligand binding to the receptor and subsequent phosphorylation of these receptors and activation of downstream pathways [45,[71], [72], [73]].

Despite the promising antitumoral activity of these molecules, some clinical trials have been performed and have not indicated an improvement in the treatment of glioma (NCT00021229, NCT00290771, NCT00423735, NCT01331291, NCT0041142, NCT00274833, NCT00014170). The causes of the negative results in these clinical trials are unclear, however, the restricted delivery of molecules across the BBB and their degradation are possible explanations [74]. In this context, the use of nanosystems for enhancing the delivery of these molecules to brain tumors and protecting them against degradation are interesting strategies [[75], [76], [77]]. The nanocarriers for the delivery of RTK inhibitors are summarized in Table 1.

**Table 1:** Nanocarriers for the delivery of RTK inhibitors.

Nanocarrier	Composition	Objective	Physical-chemical properties	Biological models	Pre-clinical outcomes	Ref
						
Liposomes	Erlotinib, doxorubicin, transferrin, cell-penetrating peptide (PFV), DOTAP, and DOPE, NHS-PEG_2000_-DSPE	Inhibit the EGFR	Particle size: 158.7–165.05 ​nm; PDI <0.232; zeta potential: 4.66 ​mV; doxorubicin EE: 65.26%; and erlotinib EE: 53.99%.	∗	Enhanced *in vitro* antitumoral activity and cellular uptake.	[78]
Polymeric nanoparticles	Gefitinib, GSK461364A, and PLGA	Inhibit the EGFR	Particle size: 101.29 ​nm; PDI: 0,102; zeta potential: 24.65 ​mV; Gefitinib EE: 26%; and GSK461364A EE: 35%	∗	*In vitro* antitumoral activity of the developed nanosystems was significantly higher than the combinations of the free drugs	[79]
Chitosan-coated lipid-core nanoparticles	Bevacizumab, gold-III, chitosan, PCL, Caprylic/capric triglyceride, and soybean lecithin	Inhibit the activation of the VEGFR signaling pathway	Particles size: 183 ​nm; PDI: 0.22; and zeta potential: 18.5 ​mV.	∗	Evidenced potent antiangiogenic activity	[80]
Polymeric nanoparticles	Imatinib, PLGA, and Pluronic® P84	Inhibit the PDGFR	Particle size: 182.63 ​nm; PDI: 0.196; zeta potential: 15.2 ​mV; and imatinib EE: 40.63%	∗	Increased the cytotoxicity effect (U251MG and C6 cells) compared with free drug	[81]

Liposomes for co-delivering doxorubicin and erlotinib, an EGFR inhibitor, were designed by Lakkadwala and Singh (2019) for glioblastoma multiforme regression. In addition to encapsulating both drugs in this lipid bilayer vesicle, the authors functionalized it with a cell-penetrating peptide (PFV) and transferrin to promote specific delivery to GBM. The encapsulation of both drugs into targeted liposomes improved the *in vitro* antitumoral activity and cellular uptake of free doxorubicin and erlotinib in GBM cells (U-87MG). Furthermore, the dual-targeted formulation was able to permeate through an *in vitro* BBB model and reach the three-dimensional cell culture, with a subsequent enhancement in the cytotoxicity effect. The results indicated the potential of targeted liposomes for delivering doxorubicin and an EGFR inhibitor [82].

Velpurisina and Rai (2019) created a polymeric nanoparticle composed of poly (lactic-*co*-glycolic) acid and polyethylene glycol for GBM treatment, co-delivering gefitinib, an EGFR inhibitor, and GSK461364A, a Polo-like kinase-1 (PLK-1) inhibitor. The authors performed an *in vitro* cytotoxicity assay of nanoparticles with different ratios of gefitinib:GSK461364A. According to the results, the higher ratio of gefitinib:GSK461364A showed a more significant synergistic killing effect in GBM cells (U-87 MG). Furthermore, the *in vitro* antitumoral activity of the developed nanosystems was significantly higher than the combinations of the free drugs, suggesting that combining two drugs in nanosystems can maximize the antitumor effect against glioblastoma multiforme [79].

Meng et al. (2020) developed nanoparticles composed of bovine serum albumin, polyethylene glycol, and poly-2-methacryloyloxyethyl phosphorylcholine for co-delivering two inhibitors of RTKs (inherbin3 and cMBP). Inherbin3 can inhibit the EGFR signaling pathway, whereas cMBP is a peptide able to block MET. The inhibition of both RTKs can contribute to a reduction in cancer progression and promote an enhancement of temozolomide chemosensitivity. The ability of the developed formulation to cross the BBB was confirmed both *in vitro* and *in vivo*. In addition, this formulation mitigated the crosstalk signaling of EGFR and MET pathways in temozolomide-resistant cells. The effect of this mitigation was also observed in the temozolomide-resistant glioma *in vivo* model, in which the combination of temozolomide with the developed formulation reduced the tumor volume 8.3 times more than the temozolomide group. The results demonstrated the potential of combining two RTK inhibitors to reduce glioma progression and enhance drug chemosensitivity [83].

Alves and colleagues (2020) designed chitosan-coated lipid-core nanocapsules functionalized with gold-III and bevacizumab (MLNC-Au-BCZ) for glioma treatment. Bevacizumab is a recombinant monoclonal antibody used to decrease tumor vasculature due to its specific binding to VEGF, which inhibits the activation of the VEGFR signaling pathway. *In vitro* studies indicated that MLNC-Au-BCZ was able to reduce cell viability by 78% at a bevacizumab concentration of 70 ​nM, while no cytotoxicity effect was observed with free bevacizumab. Moreover, the MLNC-Au-BCZ showed greater antiangiogenic activity in Chorioallantoic Membrane (CAM) assay, in comparison with free bevacizumab [80]. Another study conducted by the same research group investigated the efficacy of MLNC-Au-BCZ after pretreatment with nanocapsules functionalized with EGFRvIII peptide (MLNC-PePvIII), aiming to block EGFR and VEGFR signaling pathways. In the rat glioblastoma model, the combination of pretreatment (MLNC-PePvIII) and treatment (MLNC-Au-BCZ) resulted in a greater reduction of tumor size (4.8 ​mm^3^) when compared with the control group (37.8 ​mm^3^) and peptide and bevacizumab solution (15.6 ​mm^3^), representing a promising approach against glioblastoma [84].

The anti-angiogenic effect of bevacizumab-loaded in nanosystems in glioblastoma therapy was also evaluated by Sousa et al. (2019). The authors loaded the monoclonal antibody in polymeric nanoparticles and investigated the *in vivo* antitumoral activity of the formulation by intranasal administration in naïve mice. The nanosystem provided higher brain availability of bevacizumab (5400 ​ng/g tissue) than free bevacizumab (1346 ​ng/g tissue). Despite the bevacizumab-loaded nanoparticles having significantly reduced the tumor growth when compared with the control group, no significant difference was observed when it was compared with the free bevacizumab group. The authors attribute this result to the slow-release profile of the antibody from nanoparticles [85].

Cediranib is an RTK inhibitor that is active against VEGFR, which plays an important role in tumor growth, invasiveness, and angiogenesis. Yu et al. combined the antitumoral activity of cediranib and paclitaxel through their encapsulation in D-T7 peptide-modified pegylated bilirubin nanoparticles. D-T7 peptide was used to promote brain-targeteddelivery through its specific recognition by transferrin receptor. An *in vitro* cytotoxicity assay using C6 cells demonstrated that the co-encapsulation of cediranib and paclitaxel in the nanosystem had better cell-killing capacity than cediranib and paclitaxel alone in the nanosystem. Moreover, *in vivo* study using a glioma-bearing mice model showed an increase in the median survival rate (medium survival time of 53 days) and a decrease in tumor size when D-T7 peptide-modified co-loaded nanoparticles were compared with paclitaxel-loaded nanoparticles (medium survival time of 32 days) and cediranib-loaded nanoparticles (medium survival time of 38 days), suggesting the potential of combining a VEGFR inhibitor and a chemotherapeutic agent in a target nanosystem to improve glioma therapy [86].

Polymeric nanoparticles coated with a P-glycoprotein (P-gp) inhibitor (Pluronic® P84) were developed by Khan et al. to deliver imatinib to glioma cells overexpressing P-gp (U251MG and C6 cell lines), an efflux transporter related to multidrug resistance. Imatinib is a PDGFR inhibitor that has inhibited cell growth and proliferation in glioma. The coated nanoparticles showed greater cellular uptake (1.4-fold higher) in P-gp expressing cell line in comparison with the uncoated formulation. *In vitro* results showed that the encapsulation of imatinib into nanoparticles increased its cytotoxicity effect in both cell lines (3.2- and 2.9-fold higher in U251MG and C6 cells). In addition, imatinib-loaded coated nanoparticles increased, even more, the cytotoxicity effect when compared with the free drug (5.5- and 5.6-fold higher in U251MG and C6 cells), demonstrating the importance of inhibiting P-gp for improving the efficacy of imatinib in glioma treatment [81]. Another study carried out by Kamali et al. has also identified a greater reduction in cellular viability (1.6-fold) when imatinib was encapsulated in human serum albumin nanoparticles, which indicates the potential antitumoral effect of imatinib loaded in nanosystems [87].

#### 2.1.2 Active targeting of nanomaterials to RTK

In addition to RTK signaling pathways being targets in the treatment of gliomas and other solid tumors, the overexpression of these receptors in tumor and vascular cells makes them promising candidates for the active targeting of drug delivery nanosystems to glioma cells. Several ligands that can bind specifically with the extracellular domain of RTKs, such as cetuximab, Gint4-T aptamer, AT7 peptide, and others, have been physically or covalently bound to the surfaces of nanosystems in recent years to improve their internalization into tumor cells [[88], [89], [90], [91]]. The active Targeting Strategies of Nanocarriers for RTK are summarized in table in Table 2.

**Table 2:** Active targeting strategies of nanocarriers for RTK.

Nanocarrier	Composition	Objective	Physical-chemical properties	Biological models	Pre-clinical outcomes	Ref
Fe3O4@Au magnetic nanoparticles	Cetuximab, Fe_3_O_4_, gold	Active targeting to EGFR	Particle size: 46 ​nm and zeta potential: 11.1 ​mV	Subcutaneous xenograft model	Cetuximab improved the antitumoral efficacy of magnetic nanoparticles submitted to the simultaneous application of MFH and NIR	[88]
Polymeric nanoparticles	Panitumumab, temozolomide, PLGA, and polyvinyl alcohol	Active targeting to EGFR	Particle size: 120.5 ​nm; PDI: 0.344; zeta potential: 45.78 ​mV; and encapsulation efficiency: 52.6%.	∗	The *in vitro* results demonstrated the ability of panitumumab to improve nanoparticles' internalization in GBM cells through its recognition by EGFR	[92]
Liposomes	T7 peptide, A7R peptide, doxorubicin, vincristine, HSPC, cholesterol, DSPE-PEG_2000_	Active targeting to VEGFR-2	Particle size: 95.87 ​nm; PDI: 0.10; zeta; Doxorubicin EE: 88.4%; and vincristine EE: 86.4%	Orthotropic xenograft model	Improved the *in vivo* antitumoral activity of doxorubicin and vincristine in comparison with free drugs	[7]

DOTAP: 1,2-dioleoyl-3-trimethylammonium-propane chloride; DOPE: 1,2-dioleoyl-snglycero-3-phosphoethanolamine; NHS-PEG_2000_-DSPE: polyethyleneglycol-carbamyl distearoylphosphatidylethanolamine; EE: encapsulaion efficiency; PLGA: poly(lactic-*co*-glycolic) acid, NIR: near-infrared; MFH: magnetic fluid hyperthermia; DSPE-mPEG_2000_: 1,2-distearoyl-*sn*-glycero-3-phosphoethanolamine-N-methoxy (polyethylene glycol) (ammonium salt).

Lu and colleagues produced magnetic nanoparticles due to their ability to promote a destructive effect in glioma cells by magnetic fluid hyperthermia (MFH) and near-infrared (NIR) hyperthermia. To guarantee the specificity of this treatment, nanoparticles were functionalized with cetuximab, a monoclonal antibody with high affinity for the extracellular domain of EGFR. Functionalized nanoparticles submitted to MFH and NIR showed a 3.7-fold higher apoptosis rate in U251 ​cells than in the unmodified formulation. An *in vivo* study using a subcutaneous glioma model showed that nanoparticle functionalization with cetuximab improved the antitumoral efficacy of magnetic nanoparticles submitted to the simultaneous application of MFH and NIR. This result was correlated with the ability of cetuximab to enhance cellular uptake through receptor-dependent endocytosis and the antitumoral effect of cetuximab by blocking EGFR [93]. Cetuximab was also used to active target thermosensitive magnetic liposomes to glioma cells for hyperthermia. Cetuximab functionalization increased *in vitro* cellular uptake of magnetic nanoparticles via EGFR antibody recognition. Furthermore, the antitumoral efficacy of this formulation was confirmed during an *in vivo* study in mice orthotopic xenograft glioma model [94].

Banstola and colleagues used another monoclonal antibody (panitumumab) to promote the specific delivery of temozolomide-loaded polymeric nanoparticles to the GBM. The targeted nanoparticles had a greater cellular uptake (6.73-fold) and cytotoxicity effect in U-87MG cells (high expression of EGFR) than in the LN22 ​cell line (low EGFR expression). In addition, targeted nanoparticles were more internalized into U-87MG cells (7.8-fold) compared to the unmodified formulation. Moreover, panitumumab-modified nanoparticles showed a more pronounced cytotoxicity effect in U-87MG cells (cellular viability of 26.3% at 48 ​h and 250 ​μM) compared to free TMZ (cellular viability of 49.9% at 48 ​h and 250 ​μM) and unmodified nanoparticles (cellular viability of 39.6% at 48 ​h and 250 ​μM). The *in vitro* results demonstrated the ability of panitumumab to improve nanoparticles’ internalization in GBM cells through its recognition by EGFR, with a subsequent increase in the antitumoral activity of temozolomide-loaded nanoparticles [95].

Zhang and colleagues modified the surface of liposomes with two peptides (T7 and A7R peptides) for delivering doxorubicin and vincristine to glioma cells. T7 peptide was used to enhance liposome permeation through the BBB, whereas A7T peptide was used to promote active targeting of liposomes to glioma cells by specific recognition of A7T peptide by VEGFR-2. The authors performed an *in vitro* BBB model and investigated the ability of dual-modified liposomes to permeate and kill glioma cells (C6 line). According to the results, dual-modified liposomes improved the permeation of the drugs through the BBB and reduced C6 viability to 40.05%, a 2.55-fold higher reduction than free drugs. This formulation improved the *in vivo* antitumoral activity of doxorubicin and vincristine (tumor proliferation rate of 33.31%) in comparison with free drugs (tumor proliferation rate of 88.44%) and unmodified formulation (tumor proliferation rate of 85.31%), demonstrating the important role of active targeting in glioma treatment [96].

Targeted nanostructured lipid carriers were designed by Di Filippo and colleagues to deliver docetaxel to GBM models. Bevacizumab was used to promote active targeting of nanoparticles to the tumor microenvironment due to its ability to bind specifically to VEGF, a VEGFR ligand upregulated in glioblastoma. An *in vivo* orthotopic rat glioma model indicated that the free drug did not reduce tumor growth due to its inability to cross the BBB. By contrast, functionalized nanoparticles reduced the tumor volume by 70% when compared with the free drug. The improvement in the *in vivo* antitumoral activity of docetaxel was 1.6-fold greater when bevacizumab was functionalized in nanostructured lipid carriers than in unmodified formulation, demonstrating the potential of functionalization to deliver docetaxel to glioblastoma multiforme [97].

Monaco and colleagues functionalized polymeric nanoparticles with Gint4. T aptamer for active targeting of dactolisib, a PI3K-mTOR inhibitor, to glioblastoma multiforme through aptamer recognition by PDGFR. Gint4-T aptamer-modified nanoparticles were only internalized in U-87 MG cells (PDGFR positive), whereas the unmodified formulation was not. Furthermore, *in vivo* study indicated that Gint4. T aptamer-modified nanoparticles were able to overcome the BBB and accumulate in the tumor, while no specific signal was observed in the negative control group, suggesting the importance of targeted nanoparticles in glioblastoma treatment [91].

### 2.2 Nanomaterial for delivering PI3K/Akt inhibitors

The PI3K/Akt/mTOR pathway is activated in almost 90% of GBM cases and is related to a poor prognosis. The lipid kinase PI3K and its target, Akt, have more than 40 downstream targets and are relevant for drug action in the treatment of GBM [98]. PI3K family members are lipid kinases with multiple cellular functions that are lost in 75% of GBM cases. PI3K is classified into three classes according to structure and function. Class I is related to tumorigenesis by attenuating apoptosis and facilitating tumor invasion (Chakravarti et al., 2004; Cantley 2002). PI3K activity can be antagonized by PTEN, but the PTEN protein is mutated in approximately 30% of GBM patients, with 15% of PTEN alterations being associated with EGFR amplification [99].

Phosphorylated Akt (pAkt) is important for tumorigenesis by inhibiting apoptosis and allowing cell proliferation. pAkt phosphorylates and translocates Mouse double minute 2 homolog (MDM2) into the nucleus which down-regulates p53. Positive pAkt levels have been linked to 10-month survival in GBM patients, whereas negative pAkt levels have been linked to 14-month survival [[100], [101], [102]].

Despite the gain in PIK/Akt function, target-specific inhibitors demonstrate low clinical efficacy and adverse effects in clinical trials with GBM patients, probably due to a lack of selectivity and alteration of several downstream targets (NCT01349660 and NCT01339052). In this context, the use of nanosystems is an interesting strategy because it makes it possible to deliver one or more molecules to brain tumors in a targeted manner and protect them from degradation [[103], [104], [105], [106]]. The Nanocarriers for the delivery of RTK inhibitors are summarized in Table 3.

**Table 3:** Nanocarriers for the delivery of RTK inhibitors.

Nanocarrier	Composition	Objective	Physical-chemical properties	Biological models	Pre-clinical outcomes	Ref
Polymeric nanoparticles	miR-21 and miR-124, PEG-b-P (Gu/Hb), Ang- PEG-b-PGu	Active targeting to LRP-1 and regulated the mutant RAS/PI3K/PTEN/AKT signaling pathway	Particle size: 28.10 ​nm; PDI: 0.187; miRNA at the Gu^+^/PO_3_^−4^ ​M ratio EE: 1:1	Orthotopic xenograft model	Enhanced *in vivo* antitumor activity through inhibition of cell migratory and invasive capacity, decreased expression of pAKT, increased expression of PTEN, reduced VEGF secretion, and targeted cellular uptake	[107]
Dendrimer	PAMAM-PEGCN2-aNKG2A and 2 ​mg of PAMAM-PEG-tLyp, siRNA	Inhibition of LSINCT5-activated signaling pathways and activation of the anti-tumor immunity	Particle size: 91.32 ​± ​0,2 ​nm NP-siRNA; 90.16 ​± ​2.9 ​nm aNKNP-siRNA; 95.65 ​± ​3.1 ​nm tLypNP-siRNA; 103.42 ​± ​2.1 ​nm tLyp/aNKNP-siRNA. Zeta Potential: 7.36 ​mV NP-siRNA; 2.7 ​mV aNKNP-siRNA; 10.18 ​mV tLypNP-siRNA; 6.81 ​mV tLyp/aNKNP-siRNA.	Orthotopic xenograft model	Enhanced of anti-tumor immunity through T cells, inhibition of signaling pathways and targeting and penetrability to BBB promoted by type-1 peptides.	[108]
Nanocapsules	Lipoid ®, Solutol ®,Labrafac® WL1349, rapamycin	Inhibition of phosphorylation of mTORC1 signaling pathway	Particle size: 110 ​nm; PDI: 0.05; zeta: 5 ​mV; Rapamycin EE: 69%	∗	Selective inhibition *in vitro* (U 87 ​MG cell) of the phosphorylation of mTORC1 signaling pathway	[109]
Polymeric nanoparticles	PLGA, PVA, P80, rapamycin	Optimizing nanoparticles containing the mammalian target of rapamycin (mTOR) inhibitor	Particle size: 247 ​nm; PDI: 0.103; zeta: -11.76 ​mV; Rapamycin EE: 28.21%	∗	The *in vitro* results demonstrated the ability of the coating to enhance the internalization of nanoparticles into glioma cells (C6 lineage).	[110]
Albumin nanoparticles	LY2157299, celastrol	Inhibition of mTORC1 and TGF-β/SMAD2 signaling pathways. Active targeting to albumin-binding receptors and nAChRs	Particle size: 126.8 ​nm; zeta: 12 ​mV; LY2157299 and celastrol EE: >75%	orthotopic glioma mouse model	Targeting glioma cells with increased therapeutic efficacy by suppressing the STAT6 pathway and by inhibiting the mTOR signaling pathway.	[111]

Liu and colleagues (2021) functionalized a polymeric nanoparticle with Angiopep-2 peptide to co-deliver *anti*-miR-21 and miR-124 into the brain to treat GBM. The miRNA-loaded targeted nanoparticles present better uptake than non-targeted ones due to the binding of angiopep-2 to the lipoprotein receptor-related protein 1 (LRP-1), which is overexpressed in U-87 MG cells. Targeted nanoparticles (23% cell proliferation at 325 ​ng/ml miRNA) exhibit greater cytotoxicity than control (100% cell proliferation at 0 ​ng/ml miRNA) in U-87 MG cells. Moreover, it presented the lowest pAKT expression, reduced VEGF secretion (37%), and cell migratory and invasiveness inhibition (migration 77%, invasion 89%). The nanoparticles (t_1/2,β_ ​= ​52.7 ​min) showed longer blood circulation time than that free miRNA (t_1/2,β_ ​= ​5.6 ​min), indicating protection of miRNA from degradation. The miRNA-targeted nanoparticles showed brain uptake 2.4-fold higher than free miRNA in an orthotopic GBM xenograft model. PTEN expression increased by 32%, pAKT reduced by 72%, and increased survival by 1.2-fold relative to the PBS group showing the potential to inhibit tumorigenesis and treat GMB [104].

Kaushik and colleagues (2016) used PEG-coated gold nanoparticles and cold plasma to facilitate epithelial-mesenchymal transition (EMT) and the maintenance of cancer stem cells (CSC) in GBM using micro dielectric barrier discharge (DBD). GBM cells (T98G) were inhibited by 33% after co-treatment (100 ​nM GNP, 150 ​s plasma) and tumor sphere formation was reduced by 33%. Treatment increases ROS generation in T98G 1.5-fold which activates tumor suppressors and downregulates the PI3K/AKT pathway. Co-treatment induced p53-mediated apoptosis by activating the expression of caspase-3 and 9. The LY294002 PIK3/Akt inhibitor increased the co-treatment effect. In addition, co-treatment was able to inhibit EMT-associated transcription factors, and proteases essential for the degradation of extracellular matrix components decreased cell proliferation. Co-injection of plasma and nanoparticles resulted in 50% inhibition of tumor as compared to the control in the U-87 MG xenograft model [105].

Jin and coworkers (2020) developed a siRNA-loaded dendrimer (LSINCT5) modified with tLyp-1 peptides. The dendrimers showed high cellular uptake through multiple mechanisms (actin, caveolae, energy, and lysosomes). Glioma cells (U-87 MG) showed high cytotoxicity and an apoptosis rate of 38.94%, low migration, and poor motility. The treated cells showed significantly lower levels of p-PI3K, *p*-AKT, and *p*-mTOR. Survival was higher in dendrimer-treated U-87 MG glioma-bearing nude mice due to higher accumulation in tumor tissues after 24 ​h due to the ability of type-1 peptides to promote targeting and penetrability to the BBB. The treatment was able to enhance anti-tumor immunity through T cells such as NK cells. However, GBM inhibition occurs due to inhibition of signaling pathways and activation of anti-tumor immunity [112].

Wu and colleagues (2018) have studied the effect of zinc-doped copper oxide nanocomposites (nZn-CuO NPs) on GBM treatment. The nanocomposites inhibit proliferation in a dose-dependent manner and were more cytotoxic in GBM U-87 MG (IC50 5.7 ​μg/ml) and A172 (5 ​μg/ml) strains than in normal HUVEC (120.8 ​μg/ml) and NIH3T3 (135.6 ​μg/ml) cells. The inhibition rate on migration and invasion rate in U-87 MG cells was 75% and 89% and in A172 ​cells was 96% and 11% at 10 ​μg/ml, respectively. Nanocomposites induce ROS production and show 81% apoptosis in U-87 MG cells (at 20 ​μg/ml). They cause mitochondrial dysfunction by activating caspase-9 and caspase-3. In glioma stem-like cells (GSCs) at 20 ​μg/ml nanocomposites cause more than 80% death, while TMZ at 1000 ​μg/ml showed minimal effect on the growth of GSCs. Nanocomposites inhibit AKT and ERK1/2 activation and spheroid formation (at 10 ​μg/ml) compared to TMZ (1000 ​μg/ml) in GSCs cells. In tumor-bearing NOD/SCID mice, tumor masses were smaller in the nanocomposite-treated group than in the control group [106].

### 2.3 Nanomaterials for delivering Akt/mTOR inhibitors

In the P13K pathway, mTOR plays the role of a downstream effector and an upstream regulator through two different multiprotein complexes, mTORC1, and mTORC2. Indirect activation of mTORC1 through phosphorylation and inhibition of TSC1/2 activates phosphorylation of ribosomal protein S6 kinase (pS6k), eukaryotic initiation factor 4 ​E (eIF4E), and eukaryotic initiation factor binding protein 1 (4EBP1), involved in protein synthesis, ribosome biogenesis, and cell growth. TSC1/2 associates with and positively regulates mTORC2 by indirectly activating Akt to influence cell survival, glucose metabolism, proliferation, and cytoskeletal organization. The mTORC1 is also activated in lysosomes via amino acid signaling through Rheb or the production of phosphatidic acids by catalysis of phospholipase D1 (PLD1) translocated on lysosomes [53,113]. Some nanoparticles accumulate in lysosomes and are degraded by hydrolytic enzymes. However, some nanoparticles affect the stability of these vesicles leading to autophagy and cell death [[114], [115], [116]]. The use of nanosystems may increase the efficiency of inhibitors of these pathways in the treatment of GBM since mTOR assists in cell proliferation in GBM associated with S6K1 activation [117,118].

Escalona-Rayo and colleagues (2019) investigated the effects on C6 glioma cells of rapamycin-loaded poly (lactide-*co*-glycolide) nanoparticles coated with polysorbate 80. In vitro 2D cytotoxicity and cytoskeletal integrity, assays showed a significant increase in the efficacy of nanoparticle-based rapamycin. The nanoparticles caused >95% cell death (100 ​μg/ml), while the free drug caused 68% cell death. Significant changes in actin cytoskeleton architecture were observed in cells after 72 ​h of nanoparticle treatment compared to the free drug. The authors concluded that the changes in cell morphology suggest an apoptotic process probably due to rapamycin's ability to activate the autophagic/lysosome system through inhibition of the mTOR pathway [103]. However, we must consider that 2D cellular models do not replicate the conditions of the tumor microenvironment, an essential condition for resistance to anticancer monotherapy.

Séhédic and coworkers (2020) developed lipid nanocapsules loaded with rapamycin. Nanocapsules were more cytotoxic (IC_50_ 1 ​μM) in U-87 MG cells than free rapamycin (IC_50_ 20.54 ​μM) in normoxia (21% O_2_), and no synergistic effect was observed in association with 8Gy radiation. Rapamycin-loaded nanocapsules inhibit mTOR phosphorylation most effectively in hypoxia (0.4%). HIF-1α protein expression is reduced upon treatment with rapamycin and liposomes in either oxygenation condition, but pAkt protein level increases after treatment with nanocapsules. In the presence of 8Gy, pAkt protein expression is reduced. These results may express resistance to rapamycin due to the multiplicity of signals downstream of mTOR inhibition [119]. The loading of rapamycin, the first mTOR inhibitor, into nanoparticles improves its bioavailability and aqueous solubility, but its use is still limited by resistance in GBM. The co-delivery of rapamycin with other drugs in nanosystems is an efficient strategy to solve the problems related to monotherapy and improve its efficacy in the treatment of cancer, including GBM [47,103,119].

Liposomes for the co-delivery of honokiol and disulfiram/copper complex were developed to explore *anti*-GBM therapy via the mTOR regulatory pathway to remodel tumor metabolism and the tumor immune microenvironment. Liposomes were functionalized with a peptide (^D^CDX) to bind to nicotinic acetylcholine receptors (nAChRs). The targeted liposomes had a higher efficiency of monolayer endothelial penetration and intracellular delivery in co-cultured bEnd.3 and U-87 MG cells (nAChRs overexpressed) than the non-targeted liposomes. It showed higher antitumor efficacy (IC_50_ 0.26 ​μg/ml) than the drug combination (IC50 0.38 ​μg/ml) in U-87 MG cells. In orthotopic C6 glioma mice, survival time increased from 17 days (free drug) to 27 days (liposomes), and no pathological changes were observed in the organs. There was a 2-fold increase in the proportion of splenic CD3^+^/CD8^+^ cytotoxic T cells in the group treated with liposomes. Liposomes inhibited the Akt/mTOR signaling pathway by regulating glucose metabolism and causing death by autophagosome formation in C6 glioma [120].

Zhu and colleagues (2022) developed a biomimetic BBB-penetrating albumin nanosystem modified with a brain peptide (DCDX) to target nAChRs. The nanosystem was designed to deliver a TGF-receptor I inhibitor (LY2157299) as well as an mTOR inhibitor (celastrol) simultaneously. The nanoparticles showed higher uptake efficiency in GL261 ​cells, but penetration and uptake were 2-fold higher than non-targeted nanoparticles in a bEnd.3/GL261 ​cell co-culture system, including in tumor spheroids. The nanoparticles showed 2.9-fold higher cytotoxicity in GL261 ​cells than the inhibitor combination. The nanoparticles activated pro-apoptotic caspase-3, inhibited lactic acid secretion, and inhibited mTOR and PKM2 (a key glycolytic enzyme) pathways in glioma cells. M2 macrophage cells showed repolarization of tumor-associated macrophages (TAMs) of M2 to M1 phenotype, suppressing the STAT6 pathway. They also reduced TGF-β1 secretion and induced cell apoptosis in a co-culture of GL261 ​cells and M2 cells. In a mouse model of orthotopic glioma, the nanoparticle showed a 4.2-fold higher glioma targeting capacity than non-targeting nanoparticles, and it prolonged the survival rate 1.4-fold. The nanoparticles showed a decrease in the proportion of M2-type TAM and TGF-β1 levels in glioma tissues. The intra-glioma lactic acid concentration was 12 ​μmol/g in the drug group and was 8.4 ​μmol/g in the nanoparticle group demonstrating the decreased activity of the mTOR pathway [121].

In glioblastomas, the PKI3/Akt/mTOR pathway may have distinct and specific functions according to the survival needs of tumor cells. These characteristics should be considered when proposing therapies that inhibit PI3K pathway-mediated signals. A combination strategy designed to inhibit the PI3K/AKT/mTOR pathway is potentially effective in GBMs and GSCs. Despite inhibitory capacity the combination inhibitor therapies evaluated in clinical trials show variation in antitumor efficacy without a defined reason (NCT03696355; NCT01339052; NCT01349660; NCT02909777; NCT03522298). Therefore, the combination of inhibitors may ensure better efficacy in inhibiting important cancer cell survival pathways in GBM. While strategies using nanocarriers may decrease variability in antitumor efficacy by allowing targeted co-delivery of multiple drugs.

### 2.4 Other receptors

#### 2.4.1 P-glycoprotein

*P-*glycoprotein (P-gp) is the most common efflux pump in the brain. Also known as MDR1 and ABCD1 they are involved in the transport of a wide range of lipophilic, amphipathic xeno-, and endo-biotic substances [122]. P-gp is overexpressed in GBM cells, being the best-studied mechanism of hydrophobic anticancer drug resistance [123]. They act as an ATP-dependent drug efflux pump reducing the cellular accumulation of the chemotherapeutic drugs [124]. These receptors are also found in the blood-brain barrier (BBB), consisting of a detoxification mechanism of the brain. A synergistic approach using both P-gp substrates and P-gp modulators could be used in drug delivery nanosystems. In this way, some DDNS were developed. This strategy could improve the biopharmaceutical properties of the drugs by reducing the effective doses and improving the solubility and consequent release and bioavailability of the chemotherapeutic drugs [125,126].

The P-gp inhibitors can be classified into two types: natural and synthetics. The natural ones are glycosides, terpenes, alkaloids, flavonoids, and phenols [127]. Verapamil, Cyclosporine A, and Ketoconazole (first generation), R-verapamil, Valspodar), VX 710 (Biricodar), MS-209 (second generation), and Elacridar, Zosuquidar, and Tariquida (third generation) can all be used as P-gp inhibitors. Also, pharmaceutical excipients such as surfactants (TPGS, poloxamers, etc), polymers (polyethylene glycol, chitosan-thyobutilaminede, thiolated polycarbophil, etc), and miscellaneous (Glycerides, Miglyol, Methyl-β-cyclodextrin, etc) can be used as P-gp inhibitors [128].

The use of P-gp as a functionalizing agent in DDNS improves the cellular uptake of the DDNS by cells that overexpress P-gp, such as the glioma cells [129]. Table Topotecan liposomes modified with tamoxifen and wheat germ agglutin (WGA) were proposed as DDNS for glioma treatment. WGA is known for binding to the cerebral capillary endothelium and tamoxifen as an inhibition agent of the BBB efflux transporters. This dual-target strategy led to high inhibitory effects in C6 glioma cells. In vitro the liposomes were able to cross the BBB and target the tumor cells, presenting a better inhibitory effect when compared to the free topotecan. In vivo, the dual-target liposomes treatment improved the survival rates in brain tumor-bearing rats when compared to the free drug treatment [130]. The association of P-gp with chemotherapeutic drugs can increase the intracellular accumulation as well as enhance the efficacy of the drugs [131].

#### 2.4.2 Transferrin receptor

**Fig. 6:**
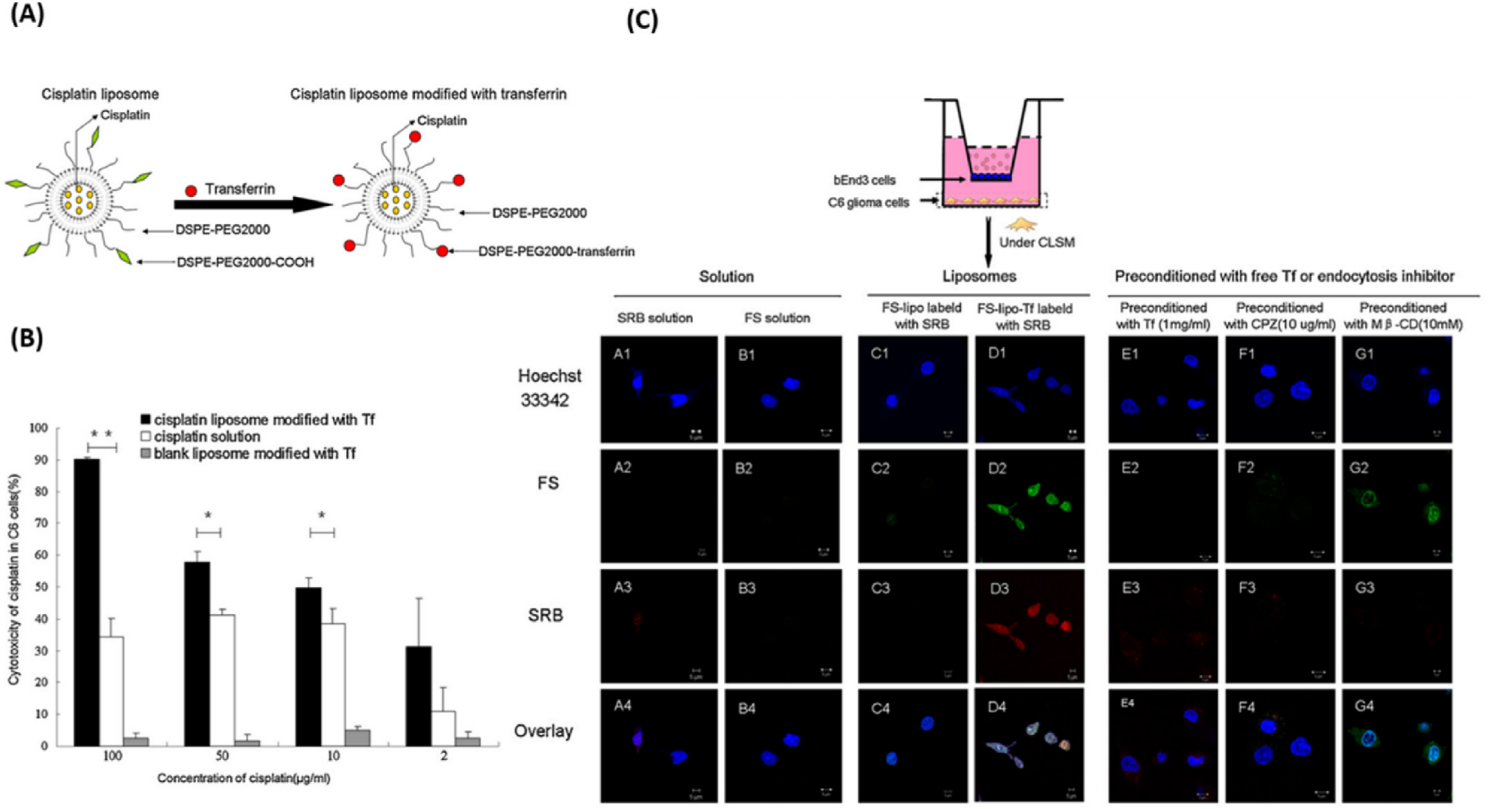
**-** (A) Schematic illustration of formation of *Cis*-lipo (Tf) nanoparticles. (B) Antiproliferative activity of cisplatin liposomes against C6 glioma cells. (C) Intracellular distribution of both FS and SRB fluorescence in C6 glioma cells after transporting across the BBB by scanning confocal microscopy. Reproduced with permission from Ref (Lv et al., 2013). Copyright 2013. Elsevier.

Transferrin (Tf) is a glycoprotein responsible for ferric ion (Fe^3+^) delivery and the remotion of toxic iron from the blood and the brain [132]. Transferrin receptors (TfR) are found in different sites of the body, such as the red blood cells and endothelial cells in the brain. Two types of TfR can be found in the body: TfR1 and TfR2. TfR1 is expressed on all cells except mature erythrocytes and terminally differentiated cells, whereas TfR2 mRNA is highly expressed in the liver and in erythroid cells, spleen, lung, muscle, prostate [133]. TfR1 is overexpressed in GBM cells, increasing iron accumulation in the cells and tumor progression [132,134]. Targeting TfR is an efficient strategy for delivering drugs to the brain (Fig. 6). Different approaches can be used to target the TfR: the endogenous ligand Tf, antibodies (e.g., OX26), peptides, and aptamers [133]. The Active Targeting Strategies of Nanocarriers for active targeting of tranferin receptor are summarized in Table 4.

**Table 4:** Active targeting strategies of nanocarriers for P-gp, transferrin, folate, integrin, and interleukin receptors.

Nanocarrier	Composition	Objective	Physical-chemical properties	Biological models	Pre-clinical outcomes	Ref
Liposomes	Topotecan, wheat germ agglutinin, EPC, PEG2000-DSPE, cholesterol, tamoxifen	Inhibit the efflux of mult drugs resistance proteins in the BBB	Particle size: 110 ​nm; PDI 0.22; zeta potential: 3.1 ​mV; topotecan EE: 85%; and tamoxifen EE: 90%.	Orthotopic xenograft model	Enhanced *in vitro* antitumoral activity and cellular uptake.	[130]
Liposomes	Cisplatin, cholesterol, DSPC, DSPE-PEG2000, protein Tf (80 ​kDa)	Target Tf receptor	Particle size: 294 ​nm; zeta potential: 1.27 ​mV; Cisplatin concentration: 478.01 μg/ml	In vitro BBB model and cytotoxicity in C6 glioma cells	The transport across the BBB was significantly increased when the cisplatin was loaded on Tf-liposomes. The cell inhibitor effect of the Tf-liposomes on C6 glioma cells was much more potent than cisplatin solution and Cisplatin liposomes	[135]
Liposomes	DOTAP, DOPE, pCMVp53	Target the Tf receptor	Particles size: 114.4 ​nm; zeta potential: 28.2 ​mV.	Orthotopic xerograph model and	The treatment with the liposomes enhanced the apoptotic response of intracranial tumors to TMZ, increase the median survival time	[136]
Dendrimers	DGL, NHS-PEG-MAL, cys-T7	Target the Tf receptor	Particle size: 141.6 ​nm; zeta potential: 3.19 ​mV	In vivo brain uptake	Quantitative analysis showed that the utilization of targeting ligand led to a 2.17-fold silencing ability, which was higher than the nanoparticles without targeting ligand, proving the qualitative results.	[137]
Red blood cell membrane-coated Solid lipid nanoparticles	Cys-NGR, cys-T7, DSPE-PEG-MAL, Glycerol monostearate, Poloxamer-188, VCR	Target the folate receptor and the CD13	Particle size: 123.6 ​nm, PDI: 0.057 ​nm and VCR EE: 55.72%	Orthotopic xenograft model	The study found that RBCSLNs modified with both T7 and NGR showed the potential to penetrate both the blood-brain barrier (BBB) and the blood-brain tumor barrier (BBTB) *in vitro*, resulting in a stronger anticancer effect compared to mono-modified RBCSLNs. The T7/NGR-RBCSLNs loading VCR demonstrated the strongest inhibitory effects by facilitating the VCR crossing the BBB and entering the glioma cells, indicating the synergistic effects of brain targeting from T7 and NGR.	[138]
Graphene oxide	Transferrin, Dox, graphene oxide sheets	Target the folate receptor	Particle size: 120.5 ​nm; PDI: 0.344; zeta potential: 45.78 ​mV; and encapsulation efficiency: 52.6%.	Orthotopic xenograft model and	The Tf-PEG-GO-Dox was the most effective in treating brain glioma in rats. The results suggest that the conjugation of Tf to the surface of PEG-GO can enhance the delivery of Dox through the blood-brain barrier and target the glioma for better treatment outcomes	[139]
Polymeric nanoparticles	PLGA, EDC, NHS, PTX, CRT	Target the folate receptor	Particle size: 118.7 ​nm; zeta: 18.3 ​mV, PTX EE: 41.4%	In vitro BBB model, biodistribution orthotopic xenograft model	CRT-NP had higher accumulation and deeper penetration in the glioma region compared to free PTX group. This might be due to the BBB-crossing ability and selective glioma penetration of CRT-NP that interact with endogenous apo-Tf. Tf-NP induced extensive necrosis and apoptotic effect to tumor cells. The tumor cell density of the CRT-NP group was significantly reduced, and the tissues were almost normalized.	[96]
Polymeric nanoparticles	TMZ, MAL-PEG2000-NHS, PAMAM, Tf	Target the folate receptor	Particle size: 118.7 ​nm; zeta: 18.3 ​mV, TMZ EE: 74.5%	Ortothopic xenograft model	PAMAM-PEG-TfR/TMZ inhibited glioma growth, induced tumor regression, and delayed recurrence in mouse models. The treatment also showed efficacy against cancer stem cells and non-stem tumor cells *in vitro* and induced apoptosis *in vivo* and in clinical samples. The nanoparticles were able to efficiently deliver drugs through the BBB via TfR targeting, consistent with previous studies.	[140]
Liposomes	Dox, DSPE-PEG2000-folate, DSPC, cholesterol, Tf	Target the folate receptor	Particle size: 180 ​nm, Dox EE: 97.5%,	Ortothopic xenograft model	Tf(F)-dox-liposome demonstrated significant tumor suppressive activity and anti-tumor effect in C6 cell-inoculated rats. It also showed a longer median survival time compared to the control groups. The H&E staining showed less abnormality in tumor sections, and liver enzyme levels were normal, indicating no adverse effects.	[141]
Transferosome	Cholesterol, TPGS-FA, DPPC, DTX	Target folate receptor	Particle size: 147.8 ​nm, PDI: 0.177, Zeta potential: 14.4 ​mV, DTX EE: 75.6%	In vitro citotoxicity in 2D and 3D models	Surface modification of transfersomes with FA can improve cellular uptake in U-87 MG cells, which can enhance the specificity of treatment for cells with FA receptor over-expression. Confocal images also showed that transfersomes were able to penetrate spheroids, which was attributed to the permeation-enhancing properties of TPGS and the deformable nature of the nanosystem.	[142]
Polymeric nanoparticles	PLGA, etoposide, DSPE-PEG, DMAB, FA, Lf	Target the folate receptor	–	*In vitro* BBB model	NPs had the highest antiproliferative efficacy compared. U87MG cells expressed a significant amount of FR and Lf/FA/PLGA NPs showed higher fluorescent intensity near these cells, indicating that the uptake of NPs by U87MG cells was through FR-mediated pathways. The targeting efficiency of NPs in delivering antitumor etoposide to brain cancer cells was strongly related to the modified FA on NPs.	[143]
Polymeric Nanoparticles	Curcumin, MPEG-PLA, FA,	Target the folate receptor	Particle size: 34.5 ​nm, PDI: 0.12, Curcumin EE: 98.5%	Ortothopic xenograft model	curcumin/Fa-PEG-PLA showed the smallest tumor fluorescence emission, and the lifetime of mice in this group was the longest, indicating that Fa-PEG-PLA improved the antitumor efficacy of curcumin.	[144]
Polymeric Nanoparticles	PEG-PBA-PEG, FA, SPION, TMZ,	Target the folate receptor	Particle size: 48.6, Zeta potential −27 mV, TMZ EE: 52.8%	In vitro antitumor efficacy	higher internalization into C6 cells and 2.5-fold higher uptake compared to unmodified NPs. The cytotoxicity of NPs on C6 cells was significantly higher than pure TMZ, with the highest apoptosis and necrosis level. There were no significant differences between the control group and blank NPs, and the blank NPs showed minimal apoptosis.	[145]
Metallic nanoparticles	FA, AuNC	Target the folate receptor	Particle size: 5.5 ​nm, PDI: 0.005	Ortothopic xenograft model	A-AuNCs were efficiently taken up by glioma cells, which can enhance their radiosensitizing efficacy. The cancer cells internalized FA-AuNCs more than normal cells. The results were consistent with a survival test in tumor-bearing rats, which showed that inhibition of cancer cell proliferation in the RT ​+ ​FA-AuNCs group was the main reason for their increased survival time.	[146]
Carbon nanosphere	Dox, F8, carbon spheres	Target the folate receptor	Particle size: 260 ​nm, PDI: 0.26, Zeta potential: 31 ​mV,	Ortothopic xenograft model	FR-positive tumor associated macrophages (TAMS) have a higher ability to take up DOX when treated with CFD. TAMs express FRs, possibly even more than the gross tumor cells, and CFD is selectively and efficiently taken up by these TAMs due to the FR-targeting ligand F8. The efficient *in vivo* effect of CFD on tumor targeting and tumor-regression ability is proven by the visual comparison of tumor sizes and respective tumor volume.	[147]
Polymeric nanoparticles	PLGA, PTX, PVA, NHS/EDC, RGD peptide	Target integrin receptors	Particle size: 197.2 ​nm, PDI: 0.192, PTX loading: 2.8%	Ortothopic xenograft model and biodistribution	intranasal (IN) administration of nanoparticles (NPs) with cancer-specific ligand RGD allows direct delivery to the brain and results in tumor cell-specific localization and retention in brain cancer. This reduces brain tumor significantly while minimizing unwanted side effects on normal tissues. The study shows that the IN inoculation of tumor-targeting RGD-NP-PTX results in effective delivery of anticancer drugs to the brain and controlled tumor growth.	[148]
Polymeric nanoparticles	PTX, PLGA, SPIONs, RGD peptide	Target integrin receptors	Particle size: 255 ​nm, PDI: 0.15, Zeta potential: 19 ​mV, PTX EE: 27%	Ortothopic xenograft model	The study found that the active ​+ ​magnetic group and magnetic group had higher accumulation of nanoparticles at the tumor site compared to other groups, as shown in contrast difference in imaging. All treated groups showed significantly less tumor progression than the control group, with median survival times ranging from 41 to 46.5 days. The active targeting group had a median survival time of 44 days, while the combination of active ​+ ​magnetic targeting had a median survival time of 45 days.	[149]
Polymeric nanoparticles	CLT1-PEG-PLA, PTX	Target integrin receptors	Particle siza: 104.7 ​nm, Zeta potential: 21.5 ​mV, PTX EE: 37.45%	Ortothopic xenograft model	The CLT1 ligand on NPs allowed for deep penetration into glioma spheroids due to its affinity for fibronectins. The CNP-PTX formulation had the best inhibitory effect on glioma growth, likely due to its better penetration and cellular uptake. The CNP-PTX group also had the most severe cell apoptosis in brain glioma tissue sections, indicating deeper penetration and more cytotoxicity compared to other treatment groups.	[150]
Polymeric nanoparticles	RGD, IL-13p, MPEG-PCL, PEG-MAL, EDC,	Target integrin receptors	Particle size: 120.1 ​nm, PDI: 0.187, Zeta potential: 9.67 ​mV	Ortothopic xenograft model	RGD modified nanoparticles (RNPs) could be internalized by the integrin receptor αvβ3, and the addition of IL-13p led to further improvement in cellular uptake and penetration ability. In vitro analysis showed that comodification of IL-13p and RGD onto nanoparticles led to the highest cellular uptake and better penetration ability. The *in vivo* study further demonstrated that tumor cell and neovasculature dual targeting could improve the localization of delivery systems in GBM site, leading to a better *anti*-GBM effect	[151]
Magnetic nanoparticles	MnFe2O4 NPs, RGD, EDC/sulfoNHS	Target integrin receptors	Particle size: 18.3 ​nm	In vitro citotoxicity	flowerlike shape NPs induced the highest expression levels of hsp70 and the highest cell death percentages, indicating their potential as effective heat mediators for hyperthermia.	[152]
Liposome	Hydrogenated soybean phosphatidylcoline, DSPE-PEG, cholesterol, Dox, AP-1 peptide	Target IL-4 receptor	–	Ortothopic xenograft model	administering AP-1 Lipo-Dox followed by sonication led to a significant colocalization of Dox with tumor cells, resulting in a higher growth inhibition compared to administering AP-1 Lipo-Dox alone. The combination of AP-1 Lipo-Dox and pulsed HIFU (high-intensity focused ultrasound) was more effective in inhibiting tumor growth and improving animal survival than either treatment alone.	[153]
Polymeric nanoparticles	PEG-PLGA, Pep-1, Poloxamer-188, coumarin	Target IL-13 ​Rα2	Particle size: 94.25 ​nm, PDI: 0.117, Zeta potential: 34.8 ​mV, Coumarin EE: 86.35%	Ortothopic xenograft model	Pep-conjugated PEG-PLGA nanoparticles were able to penetrate the tumor site more effectively than unmodified nanoparticles. This was due to the targeting effect of Pep-1, which facilitated the accumulation of particles in the glioma via IL-13Ra2 mediated endocytosis. Penetration experiments in 3D avascular C6 glioma spheroids showed that Pep-NP penetrated much deeper into the tumor tissue than unmodified NP, suggesting that it could offer a potential drug delivery carrier for glioma treatment.	[154]
Micelles	Dox, PEG-PLGA, I6P8 peptide	Target IL-6	Particle size: 24.11 ​nm, PDI: 0.121, Dox EE: 90.1%	In vitro BBB model and Ortothopic xenograft model	I6P8 peptide is important in mediating micelles across the blood-brain barrier (BBB) and accumulating within the glioma area. The I6P8-D-M treated mouse exhibited the highest glioma apoptosis compared to other groups, including the commercial TMZ treated group. Therefore, the multifunctional I6P8 peptide-linked classical PEG-PLGA micelle is a highly efficient glioma-targeted therapeutic platform.	[155]

Tf: transferrin, EPC: egg phosphatidylcholine, PEG2000-DSPE: polyethylene glycol distearoylphosphosphatidylethanolamine, DOTAP: 1,2-dioleoyl-3-trimethylammonium propane, DOPE: dioleolylphosphatidyl ethanolamine, DGL: Dendrigraft poly-l-lysine, NHS-PEG-MAL: Malemidyl-N-hydroxysuccinimidyl polyethyleneglycol, VCR: Vincristine, Dox: Doxirubicin, PLGA: Poly(d,l-lactide-*co*-glycolide), EDC: (N-(3-(dimethylamino)- propyl)-N′-ethylcarbodiimide hydrochloride), NHS: N-hydrox- ysuccinimide, PTX: paclitaxel, TMZ: temozolomide, MAL-PEG2000-NHS: maleimide-polyethylene glycol 2000-amino succinimidyl succinate, DPPC: Dipalmitoyl-phosphatidylcholine, DTX: docetaxel, DMAB: didodecyldimethylammonium bromide, FA: folic acid, Lf: lactoferrin, AuNC: Gold nanocluster, PVA: poly(vinyl alcohol), MPEG-PCL: Methoxy poly- (ethyleneglycol)-poly(ε-caprolactone).

TfR targeting liposomes can be used for the delivery of anticancer drugs. The coupling with Tf in cisplatin liposomes increased the inhibitory effects of cisplatin in C6 glioma cells by four times, being able to cross the BBB and targeting the glioma cells [135]. In another study, a cationic liposome was functionalized with the *anti*-TfR single-chain antibody fragment SGT-53 for delivery of wtp53 plasmid DNA to increase the sensitivity to temozolomide (TMZ) in TMZ resistant-cells. *In vitro,* the combination of TMZ and the liposomes increase the TMZ response to f U-87 MG and U251 GBM cell lines. *In vivo,* in a U-87 MG-luc2 xenograft tumor model, the combination of SGT-53 and TMZ leads to inhibition of tumor growth and tumor regression during the treatment. Also, the combination of SGT-53 and TMZ increased the survival by 7.5-fold compared to the TMZ alone, showing the synergistic effect of the combination [136,156].

Kuang et al. developed T7 peptide-functionalized nanoparticles for gene delivery. The T7 peptide can bind to TfR with a similar affinity as Tf. The targeted nanoparticles presented higher transfection (1.7-folds) in U-87 MG cells than un-targeted nanoparticles. Furthermore, *in vivo* study indicated that un-targeted nanoparticles had less accumulation in the glioma than the targeted nanoparticles, demonstrating that functionalization with the T7 peptide is an interesting strategy for promoting active targeting [137]. In another study, solid lipid nanoparticles (SLN) coated with red blood cell membrane, T7, and NGR peptides for the delivery of vincristine were developed. Due to the functionalization, the SLNs were more internalized by C6 glioma cells when compared to non-functionalized SLNs, being essential to improve cytotoxicity of the vincristine and increase the penetration of the BBB and the blood-brain tumor barrier (BBTB) in an *in vitro* model. *In vivo,* the SLN leads to a higher anti-glioma effect when compared to the non-functionalized SLN and the free vincristine. Also, the treatment improved the median survival days of the animals and decreased the toxicity of the vincristine [138].

Inorganic DDNS also is used for the target TfR using transferrin, including nanoscaled graphene oxide (GO) for the delivery of doxorubicin [139], and carbon dots (C-dots) for the delivery of temozolomide and epirubicin [157]. The functionalization improved the intracellular uptake of the nanoscaled graphene oxide by C6 glioma cells. *In vivo,* in a C6 glioma-bearing rat model the GO accumulated more in the glioma tissue when compared to normal brain tissue. Also, the treatment with the GO significantly decreases the tumor volume and prolonged the median survival of the animals when compared to free doxorubicin or non-functionalized GO, which highlights the potential of these systems as *anti*-GBM therapies [139].

Polymeric nanoparticles are also applied in DDNS. Kang et al. (2015) created poly (ethylene glycol)-poly (L-lactic-*co*-glycolic acid) (PLGA) nanoparticles conjugated with the CRT peptide, a peptide that mimics iron binding to the complex Tf and TfR. In an *in vitro* BBB model, the CRT increased the transport of the nanoparticles across BCEC cells, also increase the penetration in a C6 glioma spheroids model and improved the antiproliferation efficacy of C6 cells when compared to the non-functionalized nanoparticles. *In vivo* in a mouse model bearing orthotope glioma, the CRT-NP increased the median survival time of the animals, improving the efficacy of PTX [158].

Sun et al. develop PEG-PLA nanoparticles functionalized with the T12 peptide to deliver PTX. The nanoparticles (NP) presented enhanced cellular uptake by U-87 MG cells and a higher antiproliferative effect when compared to non-functionalized nanoparticles, reducing cell migration and invasion. The functionalization also improved the transcytosis across BBTB. *In vivo* in a xenograft model the treatment with functionalized NP significantly reduced the tumor volume and in an orthotopic model using U-87 MG cells the NPs increased the therapeutic effect of PTX and accumulated at the tumor site, increasing the median survival time showing the potential to target the transferrin receptor in the treatment of GBM [159].

#### 2.4.3 Folate receptor

Folate receptors (FR) are cell-surface glycoproteins receptors that mediate the cellular uptake of folate. The isoform FRα is overexpressed in brain cancer and the BBB and possesses a high affinity for folic acid, which is a necessary nutrient for the initiation and progression of cancer cells and can be converted into folate [160].

The functionalization targeting the folate receptor enhances the efficacy of traditional anticancer drugs. Allied to the improvement in efficacy the transportation of the nanosystems through the BBB is also a feature presented by the folic acid-modified systems. Several types of nanosystems are found as polymeric nanoparticles [161,162], inorganic nanoparticles [163] and liposomes [164]. The Active Targeting Strategies of Nanocarriers for active targeting of folate receptor are summarized in Table 4.

A dual modification using folic acid and cell-penetrating peptide dNP2 in liposomes for the delivery of PTX improved *in vitro* the BBB penetration, the cellular uptake, and the cytotoxicity in FR-positive C6 glioma cells when compared to non-functionalized liposomes. *In vivo,* the liposomes accumulated more in the glioma tissue of C6 glioma-bearing mice after 24 ​h of intravenous injection and strong inhibition of tumor growth and increase in survival time, showing the enhancement in the tumor-targeting due to the functionalization [165]. Similar results were observed in liposomes functionalized with folate and transferrin for the delivery of doxorubicin [141].

Luiz et al. developed folate-modified transfersomes for the delivery of docetaxel. The transfersomes improved the docetaxel activity against U-87 MG cells in 2-D and 3D cultures. Also, it was observed to have higher internalization of the folate-modified transfersomes, when compared to non-modified ones. In addition to the folate, the transfersomes possess TPGS in their composition, and the synergism of these two surfaces modifiers improves the activity of transfersomes docetaxel [142].

Monomethoxy polyethylene glycol (MPEG) and polylactic acid (PLA) nanoparticles designed for the delivery of curcumin inhibited the cell viability of the GL261 glioma cell line. The functionalization with folic acid increases the anti-glioma effect of curcumin. *In vivo* in an orthotopic GBM model, the nanoparticles decreased the tumor size in increased the survival times of mice, being better than the free curcumin and the non-functionalized nanoparticles [144]. In another work poly (lactide-*co*-glycolide) (PLGA) nanoparticles for delivery of etoposide functionalized with lactoferrin and folic acid were developed. The nanoparticles presented high permeability in an *In vitro* BBB model and increased cytotoxicity to U-87 MG cells, probably due to the active targeting [143].

Magnetite nanoparticles for the delivery of temozolomide were developed by Minaei et al. The functionalization with folic acid improved the cellular uptake of the nanoparticles by C6 cells when compared to unmodified nanoparticles. Also, it was observed higher cytotoxicity of C6 exposed to the functionalized nanoparticles when compared to the non-functionalized [145]. Another strategy using an inorganic nanoparticle was the use of a gold nanocluster functionalized with folic acid (Fa-AuNC) to improve the intracranial glioma tumors radiation therapy. *In vivo*, in a tumor-bearing C6 cell model, treatment with the FA-AUNC combined with irradiation increased cell survival and improved radiation therapy efficacy, demonstrating the potential of nanoparticle functionalization in the treatment of GBM [146].

Carbo nanotubes (CN) are also applied for the target delivery using folate. Lu et al. developed CNs associated with magnetic nanoparticles for the delivery of doxorubicin (DOX). The CNs presented the pH-sensitive release of DOX and higher internalization by U-87 MG cells, which are found within endosomes in the cytoplasm [166]. Carbon nanospheres conjugated with folate for the delivery of DOX developed by Elechalawar et al. With higher cellular uptake and accumulation in the GBM site the nanospheres enhanced the survival rate in an orthotopic tumor model and decreased the tumor size [147].

#### 2.4.4 Integrin receptors

Integrins are receptor proteins responsible for cell adhesion and involved in cell-cell and cell-microenvironment communication [167]. They play an important role in sending messages to cells as well as regulating cancer cell morphology, migration, and metastasis [168]. They are transmembrane heterodimers formed by the subunits α and β and can be classified based on the ligands' preferences into four types: collagen-, laminin- or RGD peptide-binding integrins and leucocyte-specific receptors [167].

Integrins are involved in the invasiveness and survival of glioma cells, modifying the brain microenvironment, and promoting the development of the tumor environment, contributing to cancer progression [169]. Several integrins are overexpressed in GBM and are related to poor prognosis, making them a potential specific target for the treatment of GBM [170]. The integrin subtype αvβ3 is overexpressed in GMB, being found on tumor microvessels and tumor cells [149]. This suggests that target-specific integrins in GBM could reduce tumor invasion and aggressiveness [170]. The Active Targeting Strategies of Nanocarriers for active targeting of integrins are summarized in Table 4.

Polymeric nanoparticles (PLGA) functionalized with arginyl-glycyl-aspartic tripeptide (RGD) for the nasal delivery of paclitaxel were developed by Ullah et al. RDG has been widely used for targeting nanosystems to tumor cells due to its high affinity by αvβ3. The nanoparticles were strongly associated with the C6 glioma cell line and led to cell arrest at the G2/M phase. Intranasal administration of functionalized microparticles resulted in a high accumulation of functionalized microparticles in the tumor site, compared to a low accumulation of non-functionalized nanoparticles in the brain. *In vivo* the nanoparticles reduced up to 70% of the tumor size of tumor-bearing rats compared to the free PTX, reducing the tumor volume [148]. In another study, RGD-PLGA nanoparticles were associated with magnetic nanoparticles for the delivery of PTX. The RGD improved the cellular uptake of the nanoparticles by the U-87 MG cell line and *in vivo,* the RGD-nanoparticles presented similar results to non-functionalized nanoparticles [149].

PEG-PLA nanoparticles functionalized with CLT1 peptide for the delivery of PTX were developed by Zhang et al. This peptide specifically binds to fibrin-fibronectin complexes [171]. The functionalization with CLT1 peptide resulted in high penetration of the nanoparticles in a C6 spheroid model, probably due to the interaction between the CLT1 and the fibronectins in the spheroids. *In vivo* in a C6 glioma-bearing mice model, the functionalization led to an accumulation of the nanoparticles in the tumor site and increased the median survival time [150].

PEG-PLC nanoparticles were functionalized with RGD and interleukin-13. In the C6 cell line, which expresses the receptors IL13Rα2 and αvβ3, the nanoparticles were highly internalized. The dual functionalization led to internalization by receptor-mediated endocytosis and increased the endosome scape of nanoparticles in the cells and enhanced the penetration of the nanoparticle in a C6 spheroids model. In an *in vivo* model the nanoparticles accumulated in the tumor site, being observed in both neo-vessels and GBM cells, targeting efficiently the GMB [151].

The association of physical methods with targeted integrin inorganic nanoparticles is also related to the literature [172]. Gold nanorods coated with PEG-PCL and functionalized with cyclic RGD (cRGD) for the delivery of doxorubicin (DOX) were developed by Zhong et al. The functionalization led to high internalization of the nanoparticles by U-87 MG cells when compared to non-functionalized nanoparticles and the release of the DOX was induced by near-infrared radiation (NIR). *In vivo,* in U-87 MG tumor-bearing mice the association of the nanoparticles with NIR led to a reduction in the tumor volume and an increase in the survival rate [173]. Another study found that magnetic nanoparticles functionalized with cyclo (-RGDfK) and associated with a magnetic field increased cellular uptake by U-87 MG cells via nanoparticle endocytosis and exocytosis cycles. The nanoparticles induced the generation of reactive oxygen species under an alternate magnetic field exposure, leading to cell death [152].

By active targeting the integrins system, DDNS can successfully deliver chemotherapeutic drugs, diminishing side effects, improving the cellular uptake of nanoparticles, and improving the bioactivity traditionally drugs used for the treatment of GBM. Some evidence shows that the delivery of nanoparticles targeting αvβ3-Integrin is made by neutrophils that can carry the nanoparticles to the tumor vasculature and the tumor environment [174]. The RGD-functionalized nanoparticles probably are internalized by an energy-dependent pathway, including caveolae-mediated endocytosis, clathrin-mediated endocytosis, and micropinocytosis [175].

#### 2.4.5 Interleukins

The interleukins are cytokines produced by the leukocytes and polymorphonuclear phagocytes that regulate the inflammatory process [176]. In GBM interleukins are involved in pathogenesis and malignancy. GBM tissue and microenvironment contain high levels of IL-1b, IL-6, and IL-8 and together with other substances, they influence the proliferation, invasiveness, angiogenesis, stemness, and tumor growth [177]. A wide range of interleukins are present at high levels in GBM as irterleukin-13, interleukin-4, interleukin-6 among others.

One of the main interleukin receptors overexpressed in GBM is the IL13α 2 ​R receptor, being a promising candidate for GBM immunotherapy [[178], [179], [180]]. The Active Targeting Strategies of Nanocarriers for active targeting of interleukin are summarized in Table 4. Wang et al. developed PEG-PLGA nanoparticles conjugated with Pep-1, a specific ligand of the IL13α 2 ​R. The functionalization with Pep-1 leads to high internalization by C6 cells in an energy-dependent process and high penetration in a C6 tumor spheroid model and *in vivo* accumulation in the brain, in a glioma region, making it a promising formulation for target drug delivery in GBM treatment [154]. In another work PEG-nanoparticles functionalized with Pep-1 for the delivery of paclitaxel were developed by Jiang et al. With a concentration- and energy-dependent cellular uptake the Pep-1 nanoparticles were localized in lysosomes in U-87 MG cells [181].

**Fig. 7:**
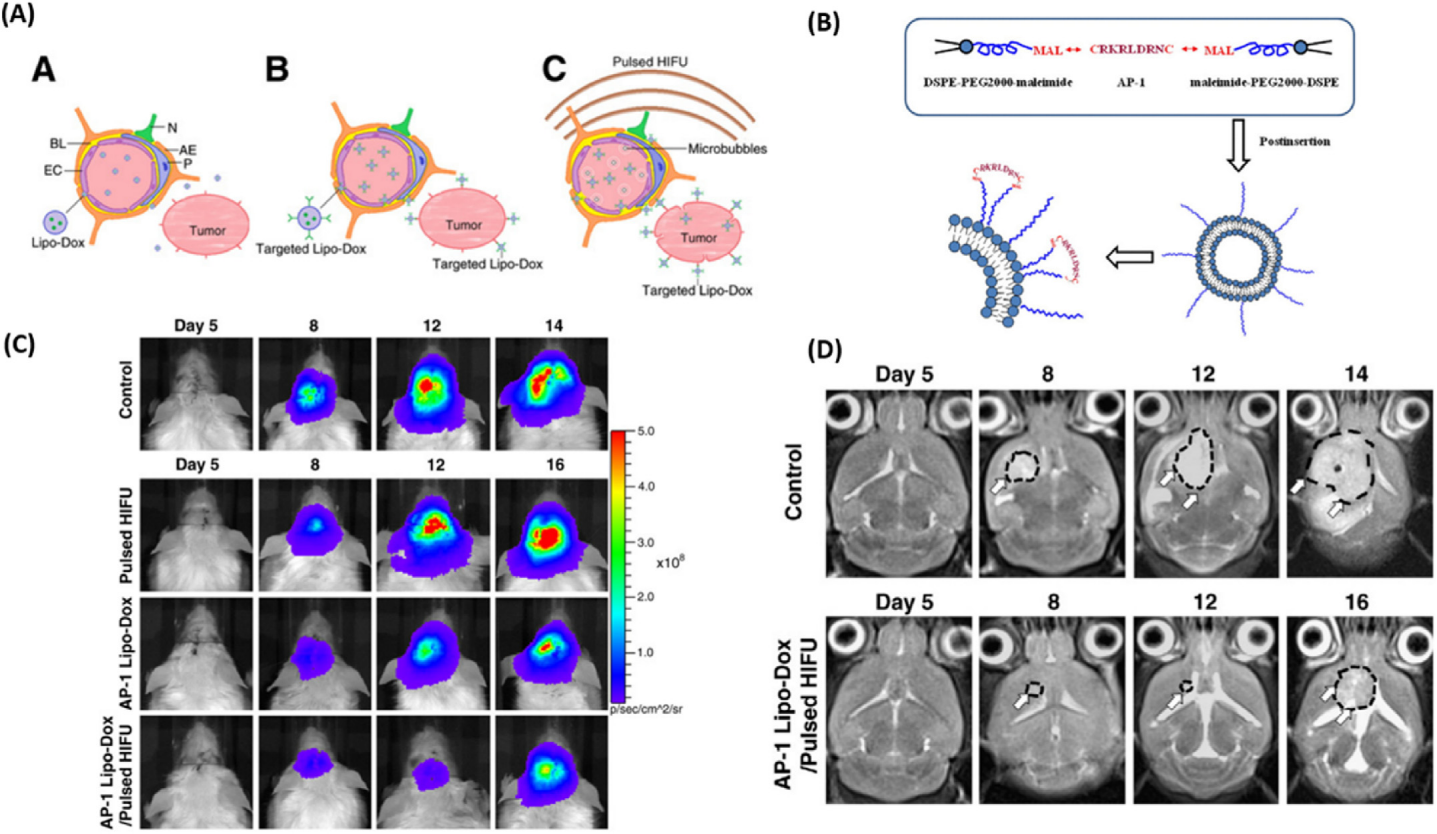
**-** (A) Schematic depiction of the treatment strategy. (B) Schematic diagram of the liposome and AP-1-conjugated liposome. (C) Biophotonic imaging of longitudinal brain tumor. (D) Representative sample of T2-weighted magnetic resonance imaging of a human GMB 8401 xenograft postimplantation. Reproduced with permission from Ref (Yang et al., 2012). Copyright 2012. Elsevier.

Another target is the interleukin-4 receptors (IL-4R), a receptor highly expressed in malignant glioma cells [182]. Liposomes functionalized with human atherosclerotic plaque-specific peptide-1 (AP-1) for the delivery of doxorubicin were developed by Yang et al. The intracranial treatment with the liposomes associated with focused ultrasound inhibited tumor growth and increased the median survival time of mice with a glioma xenograft model. The combination of active target and focused ultrasound improved doxorubicin therapeutic efficacy (Fig. 7) [153].

IL-6 is considered the first cascade-targeting ligand and its receptor (IL-6R) is highly expressed in glioma cells. PEG-PLGA nanoparticles functionalized with l_6_p_8_ peptide for the delivery of DOX were developed by Shi et al. The presence of higher concentrations of the l_6_p_8_ peptide increased the cellular uptake of the nanoparticles by U251 glioma cells. Also, the functionalization led to efficiency in targeting glioma cells and high apoptosis rates, making it more effective than the free DOX. *In vivo,* in an orthotopic model, the nanoparticles were directed to the brain, accumulating in the glioma area, increasing the survival times (49.5 days) when compared to the free DOX (33.5 days) and decreasing tumor growth exhibiting high glioma apoptosis [155].

In another study oligomers targeting IL-6R for gene delivery (ING4 tumor suppressor) were developed by Wang et al. The nanoparticles functionalized with higher gene transfection of the gene in the U-87 MG cell line as well as better penetration in a U-87 MG spheroid model, which improved nanoparticle transport across the BBB. In an *in vivo* glioma-bearing mice model the intravenous administration of the nanoparticles increased the median survival time and enhanced the BBB-crossing and glioma-targeting efficacy of ING4, decreasing the tumor volume [183].

## 3 Clearance and excretion of nanomaterials

One critical aspect that needs to be considered while developing nanomaterial-based products is their clearance and excretion from the body. Nanomaterials that are not efficiently cleared from the body can interact with cells, tissues, and organs, leading to potential toxicity concerns. Therefore, understanding the clearance and excretion of nanomaterials is crucial for the development of safe and effective nanomaterial-based products.

The clearance and excretion of nanomaterials from the body can happen via various pathways, including renal, biliary, mucociliary, and mononuclear macrophage clearance. Renal clearance is the most effective excretion pathway for small nanoparticles, but many nanomaterials are too large to be cleared efficiently through this pathway. Such nanomaterials undergo biliary excretion, where they are processed by the liver and excreted through the gastrointestinal tract. Nanomaterials trapped in mucus can also be cleared via mucociliary clearance, where they are transported to the pharynx and then swallowed. However, nanomaterials that have been internalized by mononuclear macrophages can persist for a long time, trapped within the reticuloendothelial system (RES) [184].

In general, smaller nanoparticles tend to be cleared more rapidly from the body than larger nanoparticles. This is because smaller nanoparticles can penetrate the cellular and extracellular barriers more easily, and they can be taken up by cells or eliminated through renal filtration more efficiently. On the other hand, larger nanoparticles may be retained in tissues or organs for longer periods, and they may accumulate and cause toxicity over time. The shape of nanoparticles can also affect their clearance and excretion. For instance, spherical nanoparticles are generally cleared more efficiently than rod-shaped or irregular-shaped nanoparticles, which may be more prone to aggregation and retention in tissues [185].

The surface chemistry and biocompatibility of nanomaterials can also influence their clearance and excretion [186]. Nanomaterials that have a high degree of surface charge or hydrophobicity may interact more strongly with biological membranes or proteins, leading to altered clearance and accumulation in tissues. Conversely, nanomaterials that are coated with biocompatible polymers or surface modifications, such as polyethylene glycol (PEG), can enhance their biocompatibility and reduce their uptake by immune cells, resulting in more efficient clearance and excretion [187].

## 4 Outlook

The treatment of gliomas, especially GBM, is still a challenge due to the pathophysiological characteristics of the tumor, such as the presence of BBB and the increasing resistance to currently available chemotherapeutic drugs. Another important factor to be considered is the low clinical efficacy of chemotherapeutics, with low cerebral bioavailability due to systemic biodistribution, which causes the accumulation of these drugs in non-target tissues, leading to the development of adverse effects that considerably compromise the patient's quality of life and prognosis in general.

Currently, it is known that high-grade gliomas such as GBM present several molecular alterations that lead to changes in the expression pattern of various cellular elements, such as transmembrane receptors, which are directly associated with resistance, rapid progression, and invasiveness. In this scenario, the use of nanocarriers has emerged as a tool capable of providing more effective and safer treatment for patients.

Nanocarriers can improve chemotherapeutic biodistribution by passively accumulating in tumors due to the EPR effect, which involves the accumulation of macromolecules and particles into tumors driven by the hyper-permeable vasculature and the lack of a lymphatic drainage system within tumors, has been widely accepted by the scientific community. However, recently there has been some controversy surrounding the EPR effect, as some studies have suggested that it may vary between different types of tumors. Despite this controversy, the EPR effect remains an important concept in the development of cancer therapies [188,189].

In addition to being actively targeted to the affected tissues, targeting overexpressed receptors in neoplastic cells through surface modification strategies, so that they increase the accumulation of nanocarriers in these tissues, improving drug biodistribution and brain bioavailability, and facilitating their cellular uptake through surface modification strategies. Another useful property exhibited by nanocarriers is biocompatibility modified release, being able to promote a sustained and/or triggered release (i.e.: pH-dependent release). Different nanosystems were found for blocking and/or targeting overexpressed receptors in gliomas, based on lipids and polymers, as well as dendrimers, and inorganic nanocarriers such as metallic nanoparticles.

In this review, we focused on the treatment of GBM using nanoparticles for delivering receptor blockers and active targeting of receptors overexpressed in GBM cells. However, there are new methods that can rely upon the advantages of nanoparticles to treat gliomas. For example, immunotherapy is a type of treatment that uses the immune system to fight cancer. In GBM, immunotherapy is being studied using several approaches, including checkpoint inhibitors, as discussed in this review, cancer vaccines, and chimeric antigen receptor (CAR) T-cell therapy [190]. Another important approach is gene therapy, which consists in a type of treatment that involves the delivery of genetic material to cells to replace or repair a defective gene. In GBM, gene therapy is being studied using different types of genetic materials, including the use of nanoparticles to deliver therapeutic genes to tumor cells more efficiently, since these materials are rapidly degraded by the immune system before reaching the brain [191]. Additionally, another possible approach for GBM therapy is magnetic resonance-guided focused ultrasound (MRgFUS), which is a non-invasive method that uses ultrasound waves to heat and destroys tumor cells. In GBM, MRgFUS is being studied as a potential treatment option for patients who are not eligible for surgery. In this case, magnetic nanoparticles can also be employed as a theranostic formulation, with improved tumor accumulation for image-guided diagnosis and heat-mediated apoptosis [192]. Another example of magnetic therapy for GBM is Optune™, an FDA-approved therapy based on alternating electric fields, with promising clinical outcomes [193]. Alternative administration routes can also benefit from nanoparticles’ properties, such as formulations designed for nose-to-brain drug delivery, where drugs can be directly transported into the brain parenchyma, permeating through the nasal mucosa and reaching the brain through trigeminal and vague nerves [194]. The intracranial administration after GBM resection is another important strategy being studied, where the formulation – such as hydrogels or scaffolds, is administered directly into the cranial cavity, enhancing the drug concentration into the tumor tissue, that can be further improved with the controlled release property of nanocarriers [195].

In the light of the findings in this review, the increasing importance of specific treatments for neoplastic cells and efficient tissue targeting is reinforced. Thus, the relevance of nanocarriers as tools capable of improving the biopharmaceutical properties of chemotherapeutics, with biological properties of superior efficacy and greater safety is highlighted. Although many studies emphasize these promising properties, there are still few clinical trials with nanocarriers, especially targeted ones, for the treatment of gliomas.

To achieve progress in this aspect, it is necessary to consider legal aspects, improving the legislation that regulates the manufacture, quality control, and commercialization of nanomedicines, in addition to investing in policies that favor the diffusion of these technologies to the private sector, where industries such as pharmaceutical companies can act as incubators of these technologies, improving aspects that concern the scaling and industrial production of these nanomedicines, in addition to conducting clinical trials in patients with gliomas.

## Declaration of competing interest

J.C. is a co-founder and shareholder of *TargTex S.A. Targeted Therapeutics for Glioblastoma Multiforme*. All the other authors declare no competing interests.

## Data Availability

No data was used for the research described in the article.
